# Forty years of carabid beetle research in Europe – from taxonomy, biology, ecology and population studies to bioindication, habitat assessment and conservation

**DOI:** 10.3897/zookeys.100.1523

**Published:** 2011-05-20

**Authors:** D. Johan Kotze, Pietro Brandmayr, Achille Casale, Emmanuelle Dauffy-Richard, Wouter Dekoninck, Matti J. Koivula, Gábor L. Lövei, Dietrich Mossakowski, Jinze Noordijk, Wilfried Paarmann, Roberto Pizzolotto, Pavel Saska, Axel Schwerk, José Serrano, Jan Szyszko, Angela Taboada, Hans Turin, Stephen Venn, Rikjan Vermeulen, Tullia Zetto

**Affiliations:** 1 University of Helsinki, Department of Environmental Sciences P.O. Box 65 (Biocenter 3, Viikinkaari 1), FI-00014 Helsinki, Finland; 2 University of Calabria, Department of Ecology, Ponte Bucci, I-87036 Rende (CS), Italy; 3 Università di Sassari, Dipartimento di Zoologia e Genetica Evoluzionistica, Via Muroni 25, I-07100 Sassari, Italy; 4 Cemagref UR EFNO, ‘Forest Ecosystems’, Domaine des Barres, F-45290 Nogent-sur-Vernisson, France; 5 RBINS, Entomology Department, Vautierstraat 29, B-1000 Brussels, Belgium; 6 Finnish Forest Research Institute, PO Box 18, FI-01301 Vantaa, Finland; 7 Aarhus University, Faculty of Sciences & Technology, Flakkebjerg Research Centre, DK-4200 Slagelse, Denmark; 8 Seeweg 10, D-23942 Groß Schwansee, Germany; 9 European Invertebrate Survey – Nederland, P.O. Box 9517, 2300 RA Leiden, The Netherlands; 10 Meenser Weg 9, D-37124 Rosdorf/Atzenhausen, Germany; 11 Czech University of Life Sciences in Prague, Department of Ecology, Kamycka 129, CZ-165 21 Prague 6 – Suchdol, Czech Republic; 12 Warsaw University of Life Sciences, Laboratory of Evaluation and Assessment of Natural Resources, Nowoursynowska street 166, PL-02-787 Warsaw, Poland; 13 University of Murcia, Zoology and Physical Anthropology, Facultad Veterinaria, Campus Espinardo E-30071 Murcia, Spain; 14 University of Leon, Department of Biodiversity & Environmental Management, Area of Ecology, Campus de Vegazana s/n, E-24071 Leon, Spain; 15 Loopkeverstichting (SFOC), Esdoorndreef 29, 6871 LK Renkum, The Netherlands; 16 Stichting WBBS, Kanaaldijk 36, 9409 TV Loon, The Netherlands

**Keywords:** Carabidae, ground beetle, systematics, biology, life history, rhythms, seed feeding, ant feeding, ectoparasitism, predation on amphibians, dispersal, pitfall trapping, statistics, population dynamics, long-term research, bioindicators, conservation, habitat management, landscape ecology

## Abstract

‘*Carabidologists do it all*’ (Niemelä 1996a) is a phrase with which most European carabidologists are familiar. Indeed, during the last half a century, professional and amateur entomologists have contributed enormously to our understanding of the basic biology of carabid beetles. The success of the field is in no small part due to regular European Carabidologists’ Meetings, which started in 1969 in Wijster, the Netherlands, with the 14th meeting again held in the Netherlands in 2009, celebrating the 40th anniversary of the first meeting and 50 years of long-term research in the Dwingelderveld. This paper offers a subjective summary of some of the major developments in carabidology since the 1960s. Taxonomy of the family Carabidae is now reasonably established, and the application of modern taxonomic tools has brought up several surprises like elsewhere in the animal kingdom. Progress has been made on the ultimate and proximate factors of seasonality and timing of reproduction, which only exceptionally show non-seasonality. Triggers can be linked to evolutionary events and plausibly explained by the “taxon cycle” theory. Fairly little is still known about certain feeding preferences, including granivory and ants, as well as unique life history strategies, such as ectoparasitism and predation on higher taxa. The study of carabids has been instrumental in developing metapopulation theory (even if it was termed differently). Dispersal is one of the areas intensively studied, and results show an intricate interaction between walking and flying as the major mechanisms. The ecological study of carabids is still hampered by some unresolved questions about sampling and data evaluation. It is recognised that knowledge is uneven, especially concerning larvae and species in tropical areas. By their abundance and wide distribution, carabid beetles can be useful in population studies, bioindication, conservation biology and landscape ecology. Indeed, 40 years of carabidological research have provided so much data and insights, that among insects - and arguably most other terrestrial organisms - carabid beetles are one of the most worthwhile model groups for biological studies.

## 1 Introduction

### 1.1. General

Carabid beetles are one of the best-known taxa in entomology. These beetles have been studied intensively by generations of coleopterists, who have clarified the taxonomy and phylogeny, geographic distribution, habitat associations and ecological requirements, life history strategies and adaptations, especially in Europe (e.g. Holdhaus and Lindroth 1939; Palmén 1944; Lindroth 1945a, b, 1949; Thiele 1977; Ball 1979; Desender 1986, Desender et al. 1994a; Turin 2000; Luff 2007).

This wealth of basic information has fostered a plethora of quantitative ecological studies. Indeed, the first European Carabidologists’ Meeting in Wijster, the Netherlands in 1969, touched upon one of the fascinating characteristics of carabid beetles – dispersal and dispersal power (Den Boer 1971). As a life history trait, dispersal has profound consequences for the dynamics and persistence of populations, the distribution and abundance of species and for community structure (Dieckmann et al. 1999). Not surprisingly, a summary based on the 3rd International Carabidologists’ Meeting emphasised the role of dispersal in increasingly fragmented landscapes, and argued that much more knowledge on the effects of habitat loss and fragmentation on carabid beetle population dynamics is needed if sensible decisions are to be made regarding conservation and land-use (Thacker 1996).

But why study carabid beetles? The reasons are diverse: relatively stable taxonomy, high species richness, occurrence in most terrestrial environments and geographical areas, the availability of easy collection methods, known sensitivity to environmental changes, and perceived role as beneficial in agriculture (see Darlington 1943; Lövei and Sunderland 1996; Rainio and Niemelä 2003). Armed with such a diverse wealth of knowledge, many ecologists and taxonomists have turned to carabid beetles to test ecological research questions. In this paper we emphasise progress in some of the major fields in carabidology since the first European Carabidologists’ Meeting, 40 years ago.

### 1.2. Basic knowledge

Modern disciplines in carabid beetle ecology, such as bioindication, conservation and habitat management, landscape ecology and urban ecology rely heavily on the work done by professional and amateur carabidologists from the more traditional fields of natural history, systematics and taxonomy. This species-rich family occurs in most terrestrial habitats and is found in the vegetation as well as high up in the trees and the canopy, not only in the tropics (Arndt 2005). This is probably the main reason why carabids are relatively well represented in collections around the world. In many regions, information on labels from these collections has been gathered in large databases. Combined with data from systematic sampling, such datasets enable profound faunistic work. These databases are increasingly elaborated and published as annotated checklists, red lists, catalogues and/or atlases. In combination with a clear taxonomy, mainly identification literature, these provide a sound basis for biogeographical, biological, ecological and experimental studies. Table 1 shows an overview of the major publications for the European continent, which is covered well, although there is clearly need for updating in a few regions, mainly in the east (Romania, Hungary, Russia, Caucasus). In some cases, older works are mentioned in Table 1, which belong to antiquity and do not adequately cover the fauna of that region anymore (e.g. Ganglbauer 1892; Apfelbeck 1904; Porta 1923–1959). These older works are hardly in use for identification anymore. However, they still provide historical bases for modern identification works, which often have to be elaborated from numerous smaller keys or large revisions (e.g. Jeannel 1926–28; Breuning 1932–37), such as the keys to the Carabinae (Casale et al. 1982) and to the supra-specific taxa of Italy (Casale 2005).

**Table 1.** Overview of publications concerning the faunistics of ground beetles in Europe.

*Country*	*Identification literature*	*Checklist/Catalogue*	*Atlas*
Albania	Apfelbeck 1904	Guéorguiev 2007	
Austria	Müller-Motzfeld 2004	Mandl 1972, 1978; Müller-Motzfeld 2004	
Baltic	Haberman 1968; Müller-Motzfeld 2004	Haberman 1968; Barsevskis 2003; Alexandrovitch et al. 1996	Haberman 1968
Belgium/Luxembourg	Boeken et al. 2002; Müller-Motzfeld 2004; Muilwijk et al. (In prep.)	Desender et al. 1995; 2008b	Desender et al. 2008a
Bulgaria	Apfelbeck 1904	Hieke and Wrase 1988; Guéorguiev and Guéorguiev 1995; Guéorguiev et al. 1997	
Caucasus	Iablokov-Khnzorian 1976	Kryzhanovskij et al. 1995	
Czech Republic/ Slovakia	Reitter 1908; Kult 1947; Hurka 1996	Hurka 1996, Müller-Motzfeld 2004	Skoupý 2004
Denmark	Hansen 1968; Müller-Motzfeld 2004	Bangsholt 1983	Bangsholt 1983
Fennoscandia	Lindroth 1985-1986	Lindroth 1945a, 1960, 1985-86; Strand 1970	Lindroth 1945b
France	Jeannel 1941-1942, 1949; Forel and Leplat 1995, 2001, 2003, 2005	Jeannel 1941-1942, 1949; Forel and Leplat 1995, 2001, 2003, 2005	Coulon et al. 2000; Forel and Leplat 1995, 2001, 2003, 2005; Callot and Schott 1993
Germany	Reitter 1908; Müller-Motzfeld 2004; Wachmann et al. 1995	Müller-Motzfeld 2004	Gebert 2006
Great Britain	Luff 2007	Hyman and Parsons 1992; Luff 2007	Luff 1998
Greece	Apfelbeck 1904; Arndt et al. (in press)	Arndt et al. (in press)	
Hungary	Csiki 1946	Csiki 1946	
Iberia	Forel and Leplat 1998; Herrera and Arricibita 1990; Machado 1992 (Canary Islands); Ortuño and Toribio 2005	Herrera and Arricibita 1990; Zaballos and Jeanne 1994; Serrano 2003; Machado 1992 (Canary Islands)	Herrera and Arricibita 1990; Ortuño and Toribio 2005
Iceland	Lindroth 1985, 1986; Luff 2007	Lindroth 1931; Larsson and Gigja 1959	
Ireland Italy	Anderson et al. 2000 Porta 1923-1959; Casale et al. 1982; Casale 2005	Anderson et al. 2000 Luigioni 1929; Magistretti 1965; Vigna Taglianti 1993, 2005	Anderson et al. 2000 Casale et al. 1982, 2007; CK Map 2006
Moldova/Romania	Csiki 1946	Kryzhanovskij et al. 1995; Neculiseanu and Matalin 2000	
The Netherlands	Boeken et al. 2002	Brakman 1966; Turin 2000; Muilwijk and Felix 2010	Turin 2000
Poland	Müller-Motzfeld 2004	Burakowski et al. 1973-1974; Müller-Motzfeld 2004	
Russia/Belarus	Kryzhanovskij 1983	Kryzhanovskij et al. 1995; Alexandrovitch et al. 1996	
Switzerland	Müller-Motzfeld 2004	Marggi 1992; Müller-Motzfeld 2004; Luka et al. 2009	Marggi 1992; Luka et al. 2009
Ukraine	Kryzhanovskij 1983	Kryzhanovskij et al. 1995; Putchkov 2011	
Former Yugoslavia	Apfelbeck 1904	Drovenik 1999	
Europe, general	Ganglbauer 1892; Du Chatenet 1986; Trautner and Geigenmüller 1987; Eurocarabidae: http://www.eurocarabidae.de	Turin 1981; Kryzhanovskij et al. 1995; Löbl and Smetana 2003; Fauna Europea: http://www.faunaeur.org	European maps: Du Chatenet 1986 (189 European species); Turin 2000 (380 Dutch species), Turin et al. 2003 (*Carabus*: 135 species); Fauna Europea: http://www.faunaeur.org

A sound basic list of the Carabidae of the world is the recent checklist published by Lorenz (2005) and a catalogue with distributional data is available for the Palaearctic region as a whole (Löbl and Smetana 2003). Furthermore, many recent checklists and catalogues are available (concerning Europe, see some examples in Table 1). In particular, Kryzhanovskij et al. (1995) provided detailed information on the carabid fauna of Russia and adjacent countries (including central-Asiatic). In the Western Hemisphere (the Americas), detailed information is available, especially for the regions north of Mexico (Lindroth 1961–1969; Ball and Bousquet 2001; Larochelle and Larivière 2003; Erwin 2007; Erwin and Pearson 2008), or will soon be (Erwin in preparation), but in many tropical areas of Central and South America, many genera and species remain undescribed. Other geographical areas are less well known. Asia, as a huge continent is relatively well-known in some parts, such as Siberia, Near and Middle East and especially Japan (e.g. Habu 1967, 1973, 1978), whereas immense areas are a “work in progress” (China, The Himalayas and South-East Asia). Africa is well-known in some northern countries, in particular Morocco, Algeria and Tunisia, thanks to the contributions of specialists like Antoine (1955–1962), Bedel (1899–1900) and Kocher (1963). Nevertheless, in spite of the numerous papers published by Alluaud, Basilewsky, Jeannel and others, the sub-Saharan (tropical) part of the continent needs more investigation. Australia, thanks to the C.S.I.R.O. has one of the best-organised services of insect collections, and is covered by catalogues and revisions, of which we highlight the catalogue by Lawrence et al. (1987). But also, recent investigations allowed the discovery of many new genera and species, including impressive, large sized *Pamborus* species.

Finally, remote islands and archipelagos such as like Madagascar, Papua-New Guinea and Galápagos, for instance, have been carefully investigated by specialists like Jeannel, Darlington and Desender, respectively, but produce many new discoveries every year.

In the world catalogues of (Lorenz (1998, 2005) more than 35 000 ground beetle species have been listed. An estimated number of 40 000 species, which is more than 10 times the number of described mammals, has often been mentioned (Thiele 1977; Noonan 1985). Currently, approximately 38 600 valid names occur worldwide (based on Lorenz 2005 and an estimate of approximately 100 additional new species every year). For the Western Hemisphere only, the species count currently stands at 9 374 (Terry Erwin in litt.).

More in line with the meetings are a number of thematic treatments, but again the listed works are only examples. For a more complete and thematically arranged overview of significant work in carabidology, we refer to the excellent introduction to the proceedings of the Symposium on Phylogeny and Classification of Caraboidea by Ball et al. (1998). Worth mentioning for European carabidology are the publications of the German “Gesellschaft für Angewandte Carabidologie” (GAC) with special reference to habitat studies, such as carabid beetles in river meadow habitats (GAC^ 1999^), in forests (GAC^ 2001^) and in xerothermic habitats (GAC^ 2004^). The GAC provides many carabidological papers in open access (see http://www.laufkaefer.de/gac). Other published thematic studies, often including compilations of numerous papers from various authors, concern, amongst others: biotopes (Heydemann 1962; Schjøtz-Christensen 1965), larvae (Brandmayr and Zetto Brandmayr 1982; Arndt 1991; Luff 1993), biology and periodicity (Larsson 1939), agroecology (Holland 2002), biogeography (Ball 1985; Noonan et al. 1992), dispersal ecology (Palmén 1944; Den Boer 1977; Baars 1982; Desender 1989b; Aukema 1995), morphology (Sharova 1981; Deuve 1993) and phylogeny (Ball et al. 1998). This listing is not exhaustive, especially in the fields of genetics and molecular biology, which are growing rapidly. We conclude with the classical works *Die Fennoskandischen Carabidae* (Lindroth 1945a, b, 1949, re-published in English as Lindroth 1988, 1992a, b) and *Carabid beetles in their environments* (Thiele 1977). These inspired many carabidologists and have been, for many students, the starting point of their enthusiasm.

### 1.3. European Carabidologists’ Meetings (ECMs)

In 1959, Piet den Boer, a zoologist at the Biological Station in Wijster, started pitfall trapping at several locations in the Dwingelderveld, a large area of heathland. His purpose was to test the model proposed by Andrewartha and Birch (1954), in which animal populations could be thought of as sets of smaller local populations which periodically become extinct, their sites being subsequently reoccupied. This became known (and fashionable) under the term “metapopulation” (Levins 1970). By using carabid beetles as test organisms, Den Boer was able to show that in a large area many local populations or interacting groups fluctuate in numbers of individuals in space and time, developing his theory of ‘spreading of risk’ (Den Boer 1968). According to this theory, species occupying large areas survive more easily because the reproductive success of each separate (but interacting) group differs at different places. Dispersal between these interacting groups stabilises the number of individuals in the whole population through time. Local extinctions may occur but the chances of extinction of the entire population are minimised (Den Boer 1970). Den Boer eagerly wanted to discuss this topic with other carabid beetle specialists, in particular with Carl Lindroth from Sweden, who studied the significance of dispersal and Hans-Ulrich Thiele from Germany, who studied the reproduction of these animals. Consequently in 1969, a number of eminent European carabidologists were invited to Wijster. This select group of researchers focused on the topic of dispersal and the dispersal power of carabid beetles (Fig. 1a). In 1973, Thiele invited a number of carabidologists to Rees-Grietherbush, a field station of the University of Cologne. This second ECM appeared to be an informal one and no proceedings volume was published. However, it resulted in the organisation of a now official third ECM, also at Rees-Grietherbush, by Thiele and his colleague Friedrich Weber in 1978. Most participants were German or Dutch, though Pietro Brandmayr from Italy was also present. The proceedings entitled ‘On the evolution and behaviour of carabid beetles’ was dedicated to Lindroth, who passed away in early 1979. In 1981, Weber took the initiative and organised the fourth ECM at Haus Rothenberge (Münster), on the theme ‘The synthesis of field study and laboratory experiments’. Thiele presented a lecture but his contribution for the proceedings was never received. The proceedings, dedicated to Thiele, was published after his death in 1983.

**Figure 1a.**
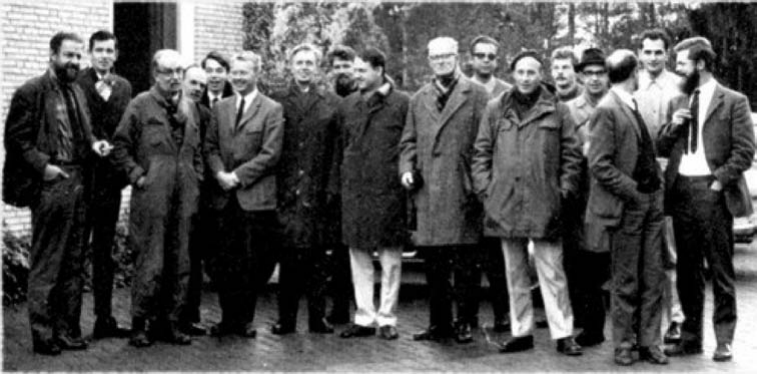
Participants of the first European Carabidologist Meeting in Wijster, 1969. From left to right: Vlijm, Van der Aart, Lindroth, Stein, Wijmans, Hengeveld, Palmén, Van Dijk, Richter, Venema, Mook, Thiele, Tjallingii, Den Boer, Haeck, Neumann, Meijer.

The first four meetings were followed by meetings organised across Europe (Table 2). As a result of political changes in Eastern Europe since the 1990s, the ECMs attained a more ‘complete’ European character. Not only did it become easier for scientists from Eastern Europe to attend these meetings, they also started to organise them. Even more noticeably during recent decades, carabidologists from beyond Europe regularly started to participate in the ECMs. Besides the official ECMs, there have been a few separate carabid beetle meetings in Europe (Table 2). Two of these (Hamburg in 1984 and Kauniainen in 1995) were not official ECM meetings, though they were mainly attended by the same carabidologists who regularly attend ECMs. The fourteen proceedings from the major ground beetle meetings that have been published before the present volume (see Fig. 1b-c, Table 2), comprise together more than 400 articles covering a wide range of topics. A rough classification of the articles leads to the following summary: Habitat preference, community ecology was the topic of 84 papers, Biology (development, preferences, etc.) of 55, Population biology - 46, Nature conservation - 35, Agro-ecology - 34, Dispersal ecology - 33, Evolutionary biology, phylogeny - 22, Morphology - 15, Ecology, general - 13, Genetics - 13, Biogeography - 11, Taxonomy - 11, Method-development - 10, Rest – 10, Faunistics - 9, and Palaeontology - 2. A similar series of meetings and proceedings started in America with the publication of the First International Symposium of Carabidology (Erwin et al. 1979). In^ 1999^, a volume consisting mainly of taxonomic papers was published, dedicated to the memory of Oleg L. Kryzhanovskij (Zamotailov and Sciaky^ 1999^).

**Figure 1b.**
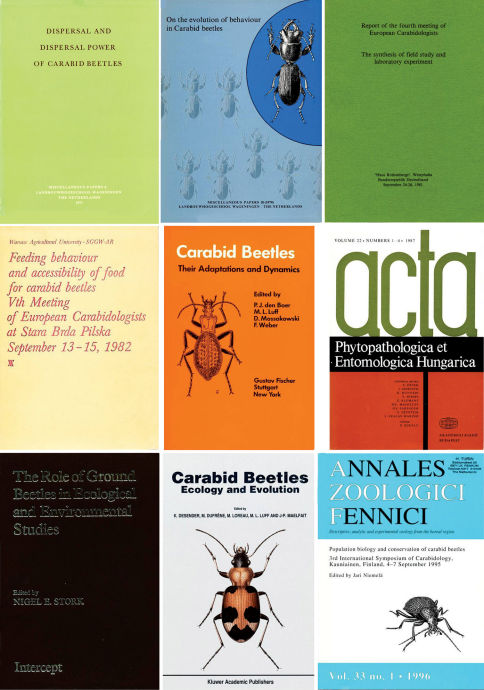
Front covers of the first European meetings, ECM 1–8 and that of Hamburg 1984 (centre cover) (see also Table 2).

**Figure 1c.**
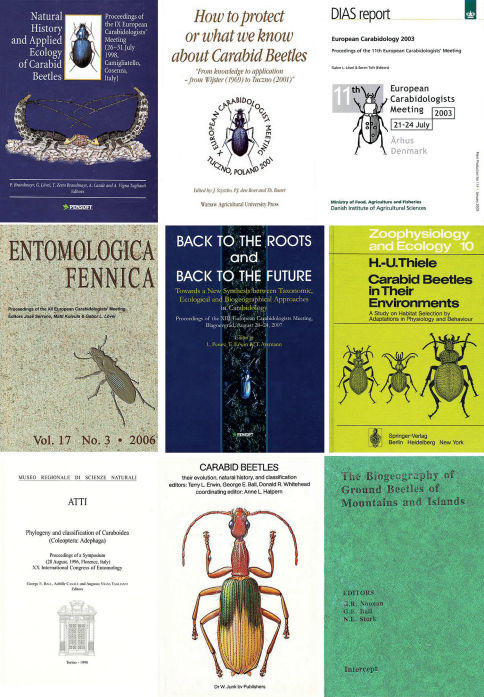
Front covers of the last five ECMs and of a few major carabidology publications (Thiele 1977; Ball et al. 1998; Erwin et al. 1979; Noonan et al. 1992) (see also Table 2).

**Table 2.** The year, location, title and editors of all the European Carabidologists’ Meetings.

*Year*	*Location*	*Proceedings*
1969	Wijster, The Netherlands (ECM 1)	1971. Dispersal and dispersal power of carabid beetles (Den Boer)
1973	Rees-Grietherbush, Germany (ECM 2)	None
1978	Rees-Grietherbush, Germany (ECM 3)	1979. On the evolution of behaviour in carabid beetles (Den Boer et al.)
1981	Münster, Germany (ECM 4)	1983. The synthesis of field study and laboratory experiments (Brandmayr et al.)
1982	Stara Brda Pilska, Poland(ECM 5)	1986a. Feeding behaviour and accessibility of food for carabid beetles (Den Boer et al.)
1984	Hamburg, Germany (17th International Entomological Congress)	1986b. Carabid beetles, their adaptations and dynamics (Den Boer et al.)
1986	Balatonalmadi, Hungary (ECM 6)	1987. Proceedings of the 6th ECM (Den Boer et al.)
1989	London, United Kingdom (ECM 7)	1990. The role of ground beetles in ecological and environmental studies (Stork)
1992	Louvain la Neuve, Belgium (ECM 8)	1994a. Carabid beetles, ecology and evolution (Desender et al.)
1995	Kauniainen, Finland (3rd International Carabidology Congress)	1996b. Population biology and conservation of carabid beetles (Niemelä)
1998	Camigliatello, Italy (ECM 9)	2000. Natural history and applied ecology of carabid beetles (Brandmayr et al.)
2001	Tuczno, Poland (ECM 10)	2002. How to protect or what we know about carabid beetles (Szyszko et al.)
2003	Århus, Denmark (ECM 11)	2005. European Carabidology 2003 (Lövei and Toft)
2005	Murcia, Spain (ECM 12)	2006. Proceedings of the XII ECM; ground beetles as a key group for biodiversity conservation studies in Europe (Serrano et al.)
2007	Blagoevgrad, Bulgaria (ECM 13)	2008. Back to the roots and back to the future. Towards a new synthesis between taxonomic, ecological and biogeographical approaches in carabidology (Penev et al.)
2009	Westerbork, Netherlands (ECM 14)	2011. Present volume (Kotze et al.)

In 2009, the 14th ECM returned to the starting grounds in the Netherlands and was attended by five participants of the first ECM: Piet den Boer, Jaap Haeck, Rob Hengeveld, Jan Meijer and Theo van Dijk. The participants visited the permanent sampling plots in the Dwingelderveld and Mantingerveld, started 50 years earlier.

## 2 Systematics, phylogeny and evolution

### 2.1. Overview

Regular carabidologists’ meetings have contributed significantly to our understanding of carabid phylogeny, evolution and systematics, as evidenced by the presentation of more than 60 papers on these topics. Progress has been made at different taxonomic ranks and in different fields of carabid systematics. At present, the integrative approach of combining morphology, molecular systematics, ethology, ecology, geographic distribution, etc., as well as the use of bioinformatics, is recognised as the best framework for solving the challenges still faced by carabidologists (Assmann et al. 2008), and by animal taxonomists in general.

What follows is a short overview of recent advances in carabid beetle systematics, concentrating on literature presented at ECMs and the international congresses mentioned above. As the main aim of this section is to present a general overview, only some of the main papers with a wide scope are cited.

### 2.2. General outline on systematics and phylogeny of the Carabidae

Ball (1979) showed that the classification of Carabidae is mostly based on morphological characters and that it includes both clade-based and grade-based criteria; classifications differ depending on the importance given to one or the other criterion. After this seminal revision, few advances have been made to unify the criteria to elect Caraboidea (splitters) or Carabidae (lumpers), and the same holds true for other high-ranked taxa. A practical synthesis of these ideas was presented by Nagel (1979a), while Ball et al. (1998) and Assmann et al. (2008) revised the issue in depth. These two last-mentioned papers highlighted the need for an integrative approach to morphology, morphometrics and molecular systematics as the appropriate way of finding rapid solutions for challenging problems.

### 2.3. Within-species diversity

An electrophoretic study on 14 Pyrenean populations of *Carabus punctatoauratus* (Assmann 1990) revealed that the Pyrenees probably hosts an isolated relict population for this species, and that bottlenecks have affected western, central and eastern populations differentially. Subtle differences at a micro-geographic scale have also been shaped by small bottleneck phenomena in this species with low dispersal power.

Range expansion of *Carabus auronitens* during the 19th century has allowed gene flow between populations in the surroundings of Münster, Germany, as evidenced by an electrophoretic study of 19 populations that showed a steep gradient of slow and fast alleles (Terlutter 1990). The high dispersal power of this species accounts for the observed allelic gradient (esterase-encoding gene) from source areas to recently colonised areas (Niehues et al. 1996). Assmann et al. (1994) showed that present-day populations of this species originated from three major refuges in southern France and that these putative core populations have contributed differentially to postglacial range expansion of the species.

Ashworth (1996) showed that Quaternary climatic oscillations did not lead to enhanced rates of extinction and speciation in carabids, as inferred from 14C-dated fossil assemblages. The future responses of Carabidae to climate change will probably be similar to that of the past, with the exception that extinction rates are expected to be higher because of human-caused habitat fragmentation.

Rasplus et al. (2000) found that populations of the threatened species *Carabus solieri* consist of two distinct clusters corresponding to subspecies *bonnetianus* and *solieri*. These populations were probably isolated during the last glaciation and are worthy of protection as gene flow is restricted between these two groups. Moreover, molecular markers suggest that the subspecies *curtii* is a hybrid between *bonnetianus* and *solieri*.

Desender et al. (2000) investigated the genetic diversity and wing polymorphism of the salt-marsh beetle *Pogonus chalceus* in 30 populations from the Atlantic coast and nine populations from the Mediterranean Basin. These Mediterranean populations showed little differentiation associated with high dispersal power, a finding possibly related to habitat instability. A higher structuring was found in Atlantic populations, which showed varying degrees of wing polymorphism and dispersal power, possibly related to adaptation to particular conditions.

Kamer et al. (2008) investigated variation in the 12S RNA sequence in populations at different geographic scales, namely the Baltic coast, inland populations across Central Europe, and Central plus Western Europe. Population structure varied as a result of complex factors that include past history and present dispersal power, amongst others. Cryptic taxa or a lack of molecular differences among siblings were also found, showing the usefulness of landscape genetic analyses.

### 2.4. Species borders and hybridisation

Koch (1986) showed that *Pterostichus nigrita* and its sibling *Pterostichus rhaeticus* are distinct species according to habitat preferences, subtle details in male and female genitalia and karyotypic numbers. Both species are reproductively isolated, as shown by crossbreeding laboratory experiments. More recently, Angus et al. (2008) described a new cryptic species in the Iberian Peninsula, *Pterostichus carri*, and a new subspecies of *Pterostichus nigrita* from Anatolia. All taxa shared a basic 2n = 36 + X male karyotype, whereas marked variation in the number of accessory chromosomes was found within and between these taxa.

Vogler and DeSalle (1994) analysed the relationships of 17 populations of *Cicindela dorsalis* along a littoral transect from New England to Veracruz. These populations are currently ascribed to four subspecies which is difficult to ascertain. Mitochondrial DNA haplotypes showed that populations could readily be grouped into two major entities that represent well defined phylogenetic species without gene flow between them, one occupying the Atlantic coast, the other inhabiting the Gulf of Mexico. Within each of these entities, moderate diversification was found but without much geographic structure, probably because of moderate gene flow between populations.

Galián et al. (1996) studied the karyotypes and the RFLPs resulting from digestion of total DNA with endonuclease EcoRI in four populations ascribed to *Ceroglossus chilensis*. Differences between these populations in terms of chromosome number and molecular data led to the conclusion that there are three cryptic species living in sympatry.

A clear distinction between *Abax parallelepipedus* and *Abax angustatus* (reported as a subspecies of the former) resulted from a morphological analysis of sympatric populations of both species, and a molecular study based on allozymes and mitochondrial DNA (Düring 2002). No molecular evidence of hybridisation between these two species was found.

Mossakowski et al. (1986) carried out a field study on the frequency of hybrids between species of the subgenus *Chrysocarabus*, *Chrysocarabus lineatus* and *Chrysocarabus splendens* in the Pyrenees. Reliable morphological characters allowed for determining the occurrence of hybrids. Both species may hybridise (up to 40% of individuals) when particular ecological conditions are met, which indicates that complete reproductive isolation has not yet been attained. However, a number of characters are fixed in each species allowing their classification as valid species. Furthermore, Düring et al. (2000, 2006) studied the mitochondrial haplotype in many *Chrysocarabus splendens* populations and found convincing evidence of introgressive hybridisation in *Chrysocarabus* (incongruence between mitochondrial and nuclear gene trees). In contrast, nuclear ITS-2 sequences showed that populations of *Chrysocarabus splendens* made up a monophyletic clade, which is sister to that made up by *Chrysocarabus lineatus* and *Chrysocarabus lateralis*. Shared haplotypes between *Chrysocarabus splendens* and *Chrysocarabus punctatoauratus* are probably the result of introgression of the latter into the former species. On the other hand, mitochondrial DNA of *Chrysocarabus rutilans* was probably acquired from *Chrysocarabus splendens* through introgression.

### 2.5. Speciation, radiation and biogeography

Juberthie (1979) analysed the evolutionary pathways of the genus *Aphaenops* (Trechinae) from putative epigean ancestors to specialised troglobionts, and noted that food must have been a major factor in promoting their morpho-functional characters. He also concluded that *Aphaenops* and other hypogean Trechinae are not living fossils but show highly derived characters, either regressive (loss of eyes and pigmentation) or positive (slender appendages, new chemoreceptors) with regard to ancestral epigean forms, with which they still share particular plesiomorphies.

Mossakowski (1979) postulated that habitat preference is an evolutionary process that can be reconstructed when matching it against a phylogenetic tree of particular taxa. He tested this hypothesis by considering the subgenus *Chrysocarabus* and concluded that there was an adaptive shift from Mediterranean to deciduous forests and a recent colonisation of alpine environments.

Liebherr (1986) constructed a phylogeny of the *Agonum extensicolle* group based on morphological quantitative characters and the allelic frequencies derived from the electrophoresis of soluble enzymes. The resulting tree was used to test the hypothesis of the vicariance effects of the Cochise filter/barrier separating the Sonoran and Chihuahuan deserts in SW North America. He argued that the zone between the deserts has probably caused vicariant events between particular pairs of species and species groups, and also between subspecies of *Agonum decorum* 6.5 to 2.8 million years ago. This barrier has probably led to the same phenomena in other carabid taxa.

Desender et al. (1990) studied speciation of the genus *Pterostichus* in the Galápagos using multivariate morphometric analysis and ecological data. They concluded that a combination of allopatric (stepping stone model) and parapatric events (segregation in altitude of two species inhabiting the same island) may explain radiation of the genus from ancestors related to *Pterostichus peruviana*, a species presently found in South America.

Andersen and Skorping (1990) presented a conclusive model of sympatric speciation of the genus *Bembidion* (and in particular in the subgenus *Chysobracteon*), in which habitat selection and the effects of parasites may give rise to disruptive selection that promotes reproductive isolation and in turn speciation. Habitat shifts in riparian carabids may have evolved in sympatry, whereas allopatry would have produced new taxa showing mere variations of the same ecological theme.

Baehr (1994) constructed a cladistic analysis of the Pseudomorphinae based on morphological characters that solved relationships of the main lineages within the subfamily. He postulated that the subfamily has an Australian-South American origin, and that it has recently spread to North America and SE Asia.

Brandmayr and Zetto Brandmayr (1994) presented an elaborated hypothesis on the evolutionary history of the genus *Abax*, based on characters of male genitalia (inflated median lobe), larval morphology, type of parental care and larval behaviour, habitat preferences and geographic distribution. Ancestors of this genus possibly inhabited lowland forests during the late Miocene, whereas most recent taxa are found in alpine grasslands and mountain forests. This suggests that there has been a major colonisation trend towards mountains during the last geological periods. A predominantly allopatric pattern was inferred for the radiation of *Abax*.

The supertribe Carabitae poses major evolutionary problems because many character states are difficult to interpret due to homoplasy, and the biogeographic patterns of tribes are not congruent at first glance with relationships derived from molecular and morphological data. A synthesis of different studies (Prüser and Mossakowski 1998; Kamer et al. 2002; Mossakowski 2002) based on the analysis of morphological characters (adults and larvae) plus molecular data, indicates that Cychrini is sister to all other tribes, and that Carabini is sister to a clade made up of tribes Ceroglossini and Pamborini. This hypothesis also postulates a Laurasian origin of Carabitae and a single migration event across the tropics. A corollary of this hypothesis is that the *Cychrus*-like mandible of *Pamborus* is a homoplasy that would result from an adaptation to feed on snails (‘cychrisation*’*).

Of the four *Calosoma* species inhabiting the Galápagos, only *Calosoma granatense* is widespread among islands and altitudinal habitats. In spite of its high dispersal power and morphological stability, this species shows substantial genetic differentiation between populations on different islands and volcanoes (Desender and Verdyck 2000). There was probably a single colonisation event from the mainland and a stepping-stone model of island colonisation. However, gene flow must have been enough to prevent speciation events. The other three *Calosoma* species of the Galápagos are endemic to localities at high altitudes on a single island, which suggests that they have originated by convergent habitat shifts.

The phylogenetic relationship of three *Carabus* species inhabiting the Tenerife and Gran Canaria (subgenus *Nesaeocarabus*) was investigated by a phylogenetic analysis based of the mitochondrial *nd5* gene (Prüser et al. 2000). The hypothesis of a close relationship between *Nesaeocarabus* and the subgenus *Eucarabus* was rejected. Instead, Canarian taxa were closely related to the subgenus *Eurycarabus* from northern Africa, southern Italy, Sardinia and Sicily. Diversification of *Nesaeocarabus* in the Canaries was congruent with the geological history of the archipelago, with a diversification of ancestors beginning 14–7 million years ago.

The subgenus *Platycarabus* includes five species living in the Alps and adjacent areas. Casale et al. (1998) tested the hypothesis of a close relationship of these species with the subgenus *Hygrocarabus*, both included in the genus *Chaetocarabus* sensu Ishikawa (1984). Separate and combined analyses of 26 adult and larval characters, and of sequences of the *nd1* gene, rejected this hypothesis, as *Platycarabus* is a robust monophyletic lineage distantly related to *Chaetocarabus*, and is even farther from *Hygrocarabus*.

Mossakowski (2005) revised the proposal of Imura (2002) of grouping the genus *Carabus* *s. l.* into 29 sections and 137 genera, based on molecular data (see also Casale and Mossakowski 2003). Analysis of the inflated median lobe of the male endophallus and the reassessment of DNA sets with stringent criteria of bootstrap values showed that (i) relationships of the subgenera of *Carabus* were poorly solved, (ii) the results do not support the hypothesis of an explosive radiation of the ancestors of this genus, and (iii) these uncertainties do not favour the ranking of subgenera to genera proposed by Imura (2002).

### 2.6. Phylogeny based on different types of characters

**Ethology**

Brandmayr and Zetto Brandmayr (1979) found that the genus *Abax* shows different stages between a pure pre-social condition of merely laying eggs with a well-developed ovipositor, and the advanced construction of a chamber, laying the eggs in capsules and taking care of brood until hatching and pigmentation of the larvae. It was concluded that behavioural characters are difficult to interpret in a phylogenetic context due to convergence. However, in some instances they provide valuable clues to reconstruct the evolution of a group and give a good phylogenetic signal.

**Morphology**

Wing folding mechanisms have been suggested to be a character with phylogenetic value at higher taxonomic ranks (Hammond 1979). Differences in the structure (presence of patches of microtrichia) and mechanism (abdominal movements helping with folding) of wing folding among lineages of Carabidae are not congruent with phylogenetic inferences derived from other characters. The Trachypachidae is a lineage distinct from carabids, a conclusion congruent with recent molecular (Maddison et al. 2009) and karyotypic data (Martínez-Navarro et al. 2011), whereas *Gehringia* was close to other carabids, as currently accepted. The basis for investigating the phylogenetic value of wing venation within Adephaga and Carabidae was outlined by Ward (1979). This topic has received little attention, perhaps because there is a generalised model in Carabidae that shows a relatively low degree of variation within particular lineages at the tribal or generic level.

Higher-ranked taxa were considered by Beutel (1998) when analysing the relationships of Trachypachidae, based on morphological and functional characters of adults and larvae. He concluded that the family Gyrinidae is sister to all other Adephagan groups. Of these clades, Haliplidae was sister to the remaining families; these were in turn split into two main clades, one made up of Carabidae (including Rhysodini and Cicindelitae), the other made up of (Trachypachidae) + (Noteridae(Amphizoidae+Dytiscidae)). These results contradict Beutel and Haas (1996), who found Trachypachinae to be sister to Carabidae; Beutel and Haas’ hypothesis has recently received support from molecular analyses (Maddison et al. 2009). Ancestors of Adephagan beetles were probably associated with riparian habitats and it has been postulated that independent colonisations of aquatic habitats gave rise to the families Gyrinidae, Haliplidae and Dytiscidae.

Liebherr and Will (1998) studied the phylogenetic value of characters of the female reproductive tract at an inclusive scale that covered the whole family Carabidae. Surprisingly no character defined the Carabidae as a monophyletic taxon; instead the Isochaeta appeared as the adelphotaxon of Anisochaeta (that included Gehringiini and Rhysodini). In turn, the Anisochaeta was divided into two clades separated by the evolution of a secondary spermatheca. Less inclusive clades within these two major groups of Anisochaeta showed relationships that agreed with previous hypotheses in some cases.

Arndt (1998) analysed the phylogenetic relationships derived from larval morphology in 44 tribes of Carabidae. He found support for a monophyletic Carabidae+Tachypachidae+Dytiscidae clade. The family Carabidae was also a monophyletic clade if Rhysodidae were excluded. The Cicindelitae was also monophyletic and showed several autapomorphies. Metriitae and Paussitae made up a monophyletic clade. The subfamily Harpalinae (“higher” carabids) appeared to be a monophyletic clade but relationships of Brachinitae were ambiguous and remain a major challenge for future studies; a close relationship with Harpalinae is unlikely.

The phylogenetic relationships among basal grade Carabidae was revisited by Kavanaugh (1998) who showed that Trachypachidae is sister to all carabid taxa examined (which confirms similar conclusions reported in former works), that the supertribe Nebriitae is a grade rather than a clade (Nebriini is separated from related tribes), and that cicindines are related to Carabini, Cychrini, Cicindelini and Omophronini.

Cladistic analyses based on different data sets (morphology, ethology, geographic distribution), were carried out to investigate the phylogeny of Paussinae (Nagel 1979b), Ozaenini plus Metriini and Paussini (Vigna Taglianti et al. 1998), the *Agra cayennensis* group (Erwin 1996), the supertribe Nebriitae (Kavanaugh 1996), the subtribe Calleidina (Lebiini; Casale 1998), the Western Hemisphere Pseudomorphini (Erwin and Geraci 2008), the tribe Rhysodini (Bell 1998; which is likely a highly specialised predator of slime moulds rather than a primitive Adephagan stock), the subfamily Broscinae (Roig-Juñent 1998), and the subfamily Psydrinae (Baehr, 1998). These studies either corroborated previous ideas about relationships of taxa or shed light on new and unsuspected hypotheses about the phylogeny and classification of taxa, including the erection of new high-ranked taxa.

**Defence substances**

Characterisation of chemical compounds used for defence and the phylogenetic interest of this trait was summarised by Moore (1979). The review showed that (i) compounds can be grouped into at least nine categories according to their chemical nature, (ii) there probably occurred a convergent development of the same substances in distantly related lineages, (iii) diversification of chemical types occurred within some subfamilies (e.g. Pterostichinae) whereas others (Harpalinae, Lebiinae) are much more uniform; (iv) the phylogenetic signal of this trait is valuable at tribal level or higher ranks; some compounds seem to vary in particular lineages (Australian Panagaeninae) and could be useful for assessing relationships at lower ranks; and (v) further insight into this trait would result from the study of biochemical synthetic pathways, fine structure of defensive glands and the detection of more subtle compounds.

**Karyotypic evolution**

A number of contributions have addressed the question on the ancestral karyotype of Adephaga and the Carabidae, and its main patterns of evolutionary change (Nettmann 1986; Serrano 1986; Serrano and Galián 1998), or referred to the karyotypic evolution of particular taxa (Harpalini: Serrano et al. 1994). The family Carabidae (915 taxa analysed) is characterised by a notable variation of the diploid number (2n = 4 - 69), the occurrence of high chromosome numbers in comparison to Polyphagan beetles, and a repeated karyotypic formula in well-studied lineages (e.g. 2n = 26 + XY in Carabini; 2n = 22 + XY in Bembidiini; 2n = 36 + X in Harpalini).

The ancestral karyotype of Coleoptera, still present in many Polyphagan lineages, 2n = 18 + Xyp, had probably undergone significant changes in the ancestors of carabids, since neither this number of autosomes nor the particular Xyp sex chromosomes are found in any carabid. The ancestral condition of a 2n = 36 + X0 male karyotype is widespread in many lineages and may be notably diversified in particular carabid lineages. The occurrence of this formula in some dytiscids and in trachypachids (Martínez-Navarro et al. 2011) provided further support to this hypothesis. However, it has not been found in lineages showing plesiomorphic morphological characters, which suggests that it has evolved rapidly in earlier offshoots of the Carabidae.

Karyotypic data have been shown to be valuable for understanding carabid systematics though it seems that karyotypic changes are not a main driving force for speciation in carabids. This is not to deny the role of karyotypic changes in reinforcing isolation mechanisms in recently originated taxa, regardless of the occurrence of speciation processes under conditions of geographic isolation or in lowland areas (Serrano 1992).

Serrano et al. (1994) summarised the karyotypic data of members of the tribe Harpalini, and found that ancestors likely had a 2n = 36 + X male karyotype. Constraints to numerical variations within this tribe are similar to those found among other carabid tribes. The Ditomina are peculiar because they show high chromosome numbers, which corroborates its ranking as a separate subtribe.

**Molecular data**

The number of molecular studies have increased since the 1990s, either based only on molecular data or (more recently) combined with other data sets. Inferred relationships have corroborated relationships derived from traditional taxonomy but also often contradicted these, thus emphasising the need of more holistic approaches aimed at obtaining robust and congruent phylogenies.

Maddison et al. (1998) published the first comprehensive DNA-based phylogeny of Carabidae. They studied the nuclear small subunit (18S) ribosomal DNA, sequenced in 35 carabid genera representing 26 tribes. All higher-level clades were monophyletic except for the Scrobifera (scaritines plus clivinines); the Trechitae was sister to Patrobines; *Morion* and Pseudomorpha were members of Harpalinae; *Psydrus* and elaphrines were sisters and both were sister to trechites plus patrobines; there was a grade including scaritines immediately below Harpalinae.

A combined analysis of larval morphological characters and molecular data of Cicindelitae showed a number of inferences that contradict current systematics: Omina had a basal position, Megacephalini was a polyphyletic taxon, and Cicindelinae was not monophyletic (Vogler and Barraclough 1998). Use of the resulting inferences showed that there are differential diversification rates among major lineages (e.g. a high rate of diversification was found at the base of megacephalines and collyrines, and another at the base of cicindelines).

Düring and Brückner (2000) investigated the phylogeny and history of lineages of Molopina using molecular analysis based on the sequence of two mitochondrial DNA fragments. Representatives of the genera *Percus*, *Molops* and *Abax* were included, as well as *Pterostichus* and *Carabus* as outgroups. These three genera made up a monophyletic clade, and *Molops* and *Abax* were sister taxa. In a further step, Brückner and Mossakowski (2006) investigated the phylogeny of the genus *Percus* by integrating previous molecular, morphological and biogeographic characters with those of nuclear 28S rRNA. This genus is likely a monophyletic taxon divided into three main clades. Relationships among the Tyrrhenian taxa remained unresolved probably as a result of recent diversification and low mutation rates of the molecular marker.

A molecular study of the tribe Harpalini based on the mitochondrial *cox1* gene (Martínez-Navarro et al. 2005) showed that (i) Pelmatellina should be included within Stenolophina, (ii) subtribe Harpalina is polyphyletic, (iii) Ditomina is a valid subtribe, and (iv) Selenophori should be ranked as a valid subtribe closely related to the Anisodactylina.

An analysis based on sequences of 28S and *wingless* genes of *Ildobates neboti* (a rare hypogean species inhabiting a few caves in eastern Spain) and related taxa showed that tribes currently included in Dryptitae (Dryptini, Galeritini and Zuphiini) made up a monophyletic clade, and that *Ildobates neboti* is a member of the Zuphiini (Ribera et al. 2006).

Vogt et al. (2005) studied the relationships of African *Anthia* and *Termophilum*, and the related *Cypholoba chaudoiri*, based on the sequence of the mitochondrial *nd5* gene. Taxa of *Anthia* made up a monophyletic clade in which *Cypholoba chaudoiri* was unexpectedly included. Taxa of *Termophilum* made up two distinct clusters, which suggests paraphyly of this genus.

Current division of the genus *Calathus* (Sphodrini) was investigated on molecular grounds by sampling a *cox1-cox2* fragment in 44 taxa (Ruiz and Serrano 2006). The monophyly of the subgenus *Calathus* was corroborated, as well as the distinctness of the monotypic subgenera *Bedelinus* and *Iberocalathus*. The subgenus *Neocalathus* is polyphyletic and needs taxonomic revision and the same holds true for the Canarian *Lauricalathus*. The latter subgenus should be divided into two subgenera, and one of these should include *Trichocalathus.*

## 3 Biology

### 3.1. Life history strategies and rhythms

Land animals evolve strategies to optimise and synchronise their life cycle with seasonal changes of the environment. For example, reproduction usually takes place under optimal conditions, while metabolism may be reduced if conditions are suboptimal (e.g. dormancy, which in carabids has thus far only been observed for larval and adult stages).

**Ultimate (limiting) factors regulating ground beetle life histories**

Ultimate factors determining beetle life cycles include variation in temperature and rainfall. Optimal development of the immature stages requires an estimated temperature range of 4–35°C. Rainfall, in combination with temperature, affects soil humidity, which is critical because eggs absorb water from their surroundings to complete embryonic development (Paarmann 1986) and larvae are sensitive to desiccation (Paarmann 1973).

Food can also be critical. Reproduction of, for example, seed-feeding carabid species may be governed by ripe seeds that usually appear at the end of the wet or warm season. Only very few habitats offer suitable conditions for polyvoltine development throughout the year, for example, lake shores, swamps and some lowland rainforests with very short dry spells.

The only ultimate factor determining carabid beetle life cycles in the Arctic, Subarctic and Antarctic, as well as in montane habitats of the temperate zone is temperature (e.g. Thiele 1977). In the summer, only a short time window exists for reproduction and development. All species in these habitats are summer developers. Species with rapid larval development, such as *Pterostichus adstrictus* (Paarmann 1994), are true summer breeders with adult hibernation only. Species with slow larval development hibernate as larvae as well as adults and require more than one season to complete their life cycle (Kaufmann 1971; Davies 1972; Matalin 2008). In the mountains of temperate Europe (altitude of 2200–2600 m) the favourable season is reduced to 3–4 months. In forest Pterostichines, especially in the large genus *Pterostichus* and in the Molopines *Abax* and *Percus*, cycles are often biennial (Brandmayr 1977). In the genus *Molops*, where embryonic development can last for more than one month and the eggs are guarded in a subterranean hole, the females disappear from the soil surface during summer, and reappear in the autumn. The subterranean larvae are active during winter, and the new generation requires a further year to reach maturity (Brandmayr and Zetto Brandmayr 1991).

Larsson (1939) recognised different reproductive strategies in carabid beetles of the temperate zone by studying museum material. He divided them into Frühlingstiere (spring breeders) and Herbsttiere (autumn breeders). Spring breeders reproduce during the spring and hibernate as adults only. Autumn breeders reproduce during the autumn and hibernate mainly as larvae. In a number of species, adults may hibernate after reproduction to enter a second reproductive period (Gilbert 1956; Vlijm et al. 1968; Schjøtz-Christensen 1968; Krehan 1970). (Schjøtz-Christensen (1965, 1966) showed that in some *Harpalus* species spring and autumn breeding populations co-occure in the same habitat. Other examples include *Abax parallelepipedus* (Löser 1970), *Poecilus lepidus* (Paarmann 1990), *Pseudophonus rufipes* (Matalin 1997a) and *Harpalus affinis* (Matalin 1998). A third breeding category – spring-autumn breeder (Matalin 1997b) – is found in the genus *Stenolophus*. In 1990 Den Boer and Den Boer-Daanje, summarising the life history strategies of 68 common carabid beetles in Drenthe (the Netherlands), found a continuum of reproduction from early spring to late autumn, and seven of them reproduced during winter. Den Boer and Den Boer-Daanje distinguished species with summer larvae (summer developers, 40 species) and species with winter larvae (winter developers, 28 species). Drenthe is located in an area with Atlantic climate: warm winters and wet summers, thus offering a broad reproductive window. In areas with a continental climate, however, this window is much narrower.

Cave environments are buffered against climatic variation and can have (i) a constant temperature throughout the year, or (ii) distinct seasonality. Trechines living in caves are mostly autumnal reproducers with winter larvae. The rhythms of *Aphaenops* and related genera may show distinct seasonality at least in the activity of adults, influenced by the cave’s air humidity (Juberthie 1969), and sometimes with two distinct annual peaks (Cabidoche 1963, 1966). Reproduction may coincide with a peak in food, as found between *Neaphenops tellkampfi* and the eggs of the orthopteran *Hadenoecus subterraneus* (Kane et al. 1975).

The seven winter breeding species found in the Netherlands (see above) connect the carabid fauna of the temperate zone with the life history strategy typical for the subtropics with winter rain. In Palestine, Bodenheimer (1934) only caught beetles from October to June. Winter breeding (rainy season breeding) is a typical reproductive strategy in habitats that are dry in the summer, such as North Africa (Paarmann 1970, 1975). In specific habitats with moist soil during the dry summer period, propagation and reproduction occur throughout the year (Paarmann 1975, 1976d). *Thermophilum sexmaculatum* and *Graphipterus serrator*, with specialised larvae that feed on ants and their brood, reproduce in the summer (Paarmann 1985; Paarmann et al. 1986; Dinter et al. 2002), but only in sandy soil that acts as a moisture trap.

In Mediterranean Europe, which is also dry in the summer, some seed-feeding carabids - the ditomines *Carterus calydonius*, *Ditomus clypeatus*, and harpaline carabid beetles that provide *Daucus* or *Plantago* seeds to their larvae (Brandmayr and Zetto Brandmayr 1974; Schremmer 1960) - show summer reproduction. Other seed-feeders (*Ophonus*, *Pseudoophonus*) are adapted to more humid soils and normally reproduce in the autumn (winter larvae; Zetto Brandmayr 1983a, b).

No information is available on the reproductive strategies of Carabinae from the subtropics with summer rain. However, it seems reasonable to suggest that they show rainy season propagation (summer breeding) in habitats which are dry during winter. A number of studies on carabid beetle life histories are available from the tropics. In Central Africa (Kivu district), which is characterised by low variation in median air temperature (0.9 °C) and low rainfall from June-August (Walter and Lieth 1960), the majority of species avoid reproduction during and around the dry season (Paarmann 1976b). Dry season propagation was only found in two species, one living in a swamp and one in a cultivated area. North Sulawesi (Indonesia) is without a dry period, yet the appearance of gonad dormancies was widespread among 155 carabid beetle species: 65% had at least one dormant female (Paarmann and Stork 1987; Stork and Paarmann 1992). Females of the canopy dweller *Colpodes buchanani* also synchronise reproduction with annual temperature changes typical of the subtropical climate (Paarmann and Paarmann 1997).

Along the Amazon River in Brazil, forests are often inundated for up to seven months of the year. This flooding is independent of the rainy season in central Amazonia. During flooding, carabid beetles occur on tree trunks or in the canopy in the inundated site, reproducing when the water level is low (Adis et al. 1986; Adis et al. 1990). In lowland rainforests, carabids aggregate in areas with an accumulated amount of organic matter, such as fruit falls (Erwin 1979b). These fruit falls are unpredictable in space and time, lasting only for a few weeks. Fig fruit falls play an important role in these rainforests, as they occur virtually throughout the year. Distinct carabid assemblages have been found at fig fruit falls in lowland rainforests of the Amazon basin (Paarmann et al. 2001), Brunei (Borcherding et al. 2000), Australia and Africa (Paarmann et al. 2006). Female gonad maturation starts immediately after locating a fruit fall, with some females carrying ripe eggs combined with the undeveloped ovaries. These ‘transport eggs’ can be deposited directly after arrival at the fruit fall, providing larvae more time for development. While moving between patches of fruit fall, females experience short gonad dormancy induced by food shortages (Paarmann et al. 2001; Arndt and Kirmse 2002).

**Proximate factors and endogenous rhythms**

During unstable temperatures, soil humidity and resources, proximate factors and endogenous rhythms play a major role in controlling carabid beetle life cycles. At temperate latitudes, many species, especially species with summer larvae, use photoperiodic changes to synchronise gonad maturation (Thiele 1977). Autumn breeding species display thermic parapause (Müller 1970): an obligatory dormancy at a genetically fixed developmental stage, where the phase of induction cannot be recognised. Larval development can only be completed after passing a certain period of time at low temperatures. Larvae of other species with winter larvae, such as *Abax ovalis* and *Abax parallelepipedus*, only pass a thermic quiescence (Müller 1970): a facultative delay or suspension of development. This may also be the case for species with winter larvae at higher latitudes (and montane regions): Subarctic populations of *Pterostichus nigrita* were still under photoperiodic control in terms of gonad maturation, yet displayed a shift of the response curve to longer day lengths (Ferenz 1975).

Annual day length amplitudes decrease from higher latitudes to the equator, as does the importance of photoperiodic changes as a proximate factor. However, day length changes of 1 h can control imaginal diapause (Norris 1959, 1965). Two carabid species from North Africa synchronise their life cycle with annual rainfall, triggered by a decrease in temperature and a decline in the photoperiod (Paarmann 1974, 1976c). This control mechanism in a rainy season breeder (or winter breeder) of the subtropics with winter rain shows marked similarities with temperate autumn breeders and aestivation (Thiele 1977).

In the Kivu region, Central Africa (see Paarmann 1976b), the maximum change in daylight is 16 min only, and the maximum annual temperature change is 0.9°C. Under such climatic conditions, temperature plays a role as a proximate factor. The temperature of the upper soil layers and the soil surface is influenced by the water content of the soil. With water loss in the upper soil layers, daily temperature fluctuations increase. Some hours of higher temperatures per day induce gonad dormancy. With the onset of rainfall, temperature fluctuations decline and dormancy is terminated. Synchronised maturation is stimulated by the increase in average temperatures (Paarmann 1986).

**Endogenous control of gonad dormancies**

The synchronisation of gonad maturation with seasonal change in ultimate factors is possible only if proximate factors influence the endocrine system controlling this maturation. Emmerich and Thiele (1969) and Hoffmann (1969) were the first to study the hormonal control of gonad maturation in spring breeders. They found a connection between proximate factors, neurosecretions and the activity of the *corpora allata*, which produces juvenile hormones (JH). JHs are necessary to complete gonad maturation in males (Ferenz and Hölters 1975). In females, only previtellogenesis is controlled by JHs. To complete ovarian maturation, the production of a second hormone is postulated. Applications of JHs to dormant beetles of the winter breeder *Orthomus barbarus* have confirmed a similar control mechanism for this breeding type (Paarmann 1976a). The same application to dormant beetles of the summer breeder *Pogonus chalceus* resulted in complete maturation of both sexes, even complete gonad maturation in females, meaning that either complete maturation is controlled by JHs only, or high temperatures suppress only the production of JHs but not of vitellogenic hormones.

Endogenous rhythms are involved in gonad maturation. Under constant environmental conditions gonad maturation is controlled by an endogenous rhythm, synchronised by an external cue such as soil temperature (Paarmann 1986). In the desert-dwelling carabid beetle *Thermophilum sexmaculatum* thermoregulational behaviour is controlled by a circannual rhythm, resulting in lower body temperatures at the end of the optimal reproductive period, which causes an inactive stage of the gonads (Erbeling and Paarmann 1986).

As part of the taxon pulse theory (Erwin 1979b), ground beetles from tropical areas undergo latitudinal and altitudinal expansion, leading to climatic specialisation, including the development of dormancy to survive unfavourable climatic conditions. If all carabid beetle dormancies are based on a uniform hormonal system, manifold convergent evolution is possible. The use of gonad dormancies to synchronise life cycles with changing environmental conditions is widespread among tropical carabid beetles. Only one *Abacetus* species, living under stable humidity and temperature conditions (the shore of Lake Kivu, Central Africa), seems to develop without dormancy. With the exception of short gonad dormancies, triggered by food shortages in the seed-feeding guild, all studied gonad dormancies are under the control of temperature as a proximate factor.

Specialisation along riparian habitats (pathway i) leads to a synchronisation of the life cycle with seasons with stable moisture conditions, especially along riverbanks. Specialisation in seasonally dry habitats (pathway ii) leads to a synchronisation of the life cycle with the period of optimal soil humidity, e.g. rainy season propagation (Paarmann 1979). While larvae of winter breeders in the subtropics with winter rainfall are adapted to comparable temperatures, a small group requires high temperatures for successful development. These specialists, whose larvae feed on ants and ant brood, have evolved along pathway (ii) in the subtropics with summer rainfall and spending the winter in gonad dormancy. Such species have yet to be reported in the temperate zone.

Larsson (1939) found no autumn breeders among 21 studied species of the old genus *Agonum*. These species are possibly all descendants of one common ancestor that reached the temperate zone along pathway (i) after which some descendant species adapted to non-riparian habitats. One member of this group, namely *Platynus* (*Agonum*, *Limodromus*) *assimilis* is a spring breeder, but its gonad dormancy is controlled in a fundamentally different way than in other spring breeders, by a photoperiodic quiescence (Neudecker and Thiele 1974).

Gaps in our current understanding of carabid beetle life history strategies include (i) a lack of knowledge on life history strategies in the subtropics with summer rainfall, in the tropics with long dry seasons and in areas with unpredictable rainfall, (ii) whether canopy dwelling carabid beetles in tropical rainforests display seasonal patterns, and (iii) a detailed study on the hormonal control of dormancies in carabid beetles, as no such studies have been performed since Ferenz (1977).

### 3.2. Carabid beetle food

Carabid beetles are generally considered polyphagous predators. However, in line with their enormous species richness and diversity in body shapes and biotopes they inhabit, a whole range of trophic specialisations occurs in the Carabidae (Hengeveld 1980a; Zetto Brandmayr et al. 1998b). Although carabid feeding ecology and biology has been studied frequently (also during ECM meetings), it is surprising how many basic questions on carabid food remain unanswered. Except for Larochelle (1990), who mentioned food preferences of 1054, mainly North-American, European and Japanese species, basic information on food preferences or requirements is often lacking, even for many common species. This chapter does not attempt to review all trophic specialisations of Carabidae; it has been done before (Thiele 1977; Hengeveld 1980a; Toft and Bilde 2002). Instead, it focuses on recent advances in the domains of seed and ant feeding, as well as unique life history strategies, such as ectoparasitism and the predation of amphibians.

**Seed feeding**

Carabid beetles accept a variety of plant foods such as leaves, fruits, pollen, seeds and fungi (Toft and Bilde 2002 and references therein). Seed feeding, or granivory, occurs in many species including polyphagous ones that prefer animal prey (Lund and Turpin 1977; Hengeveld 1980b; Toft and Bilde 2002). True granivory, i.e. where seeds are central to the species’ food budget, has evolved in two tribes of Carabidae, Zabrini and Harpalini. The ecology of granivorous carabids is of great interest since granivory required the evolution of morphological, physiological and behavioural adaptations associated with crushing, digesting and foraging for seeds. To crush hard seeds, adults and larvae of granivorous species have evolved broad mandibles with massive adductors (Zetto Brandmayr et al. 1998b; Paarmann et al. 2006). Sclerotised structures in the adult proventriculus are then used for fine grinding of the ingested seed fragments (Evans and Forsythe 1985). Behavioural adaptations have involved, for example, climbing plants and storing seeds in burrows (Thiele 1977). Physiological adaptations to seed feeding are understudied but recent evidence shows that digestion of seeds is facilitated by endosymbionts (Lundgren and Lehman 2010).

The amount of seeds eaten by carabids in the field may be substantial. Based on seed losses of artificially exposed seeds, Honek et al. (2003) estimated that up to 4000 seeds m-2 d-1 may be removed by carabid beetles in arable fields in the Czech Republic. Honek et al. (2005) reported that carabids, mainly *Amara montivaga*, destroyed about 83–88% of the annual seed production of *Taraxacum officinale* spp. agg., and Kjellsson (1985) showed that approximately 65% of the annual seed production of *Carex pilulifera* L. was consumed by a single species, *Harpalus solitaris*. However, individual capacity for eating seeds varies with season (Honek et al. 2006) as a result of natural phenological changes (transition from dormancy to reproduction, dispersal, breeding and searching for overwintering sites). Consumption is also affected by temperature (Saska et al. 2010). Clearly, carabid beetles may have an important impact on the reproductive success and dispersal of plant species, but more research is needed on how these affect the population dynamics of plants in the longer term. Larvae should also be considered in these studies, as their consumption of seeds can be comparable to that of adults (Klimeš and Saska 2010).

The consumption of particular seed species is ultimately determined by the preferences of the carabids in question. During the last 30 years, a number of authors have investigated carabid preferences for seeds in the laboratory using choice (cafeteria) experiments (Lund and Turpin 1977; Brust and House 1988; Jørgensen and Toft 1997a). Most studies, however, have established preferences based on a limited number of seed species (usually 2–5). Only Honek et al. (2003, 2006, 2007) tested seed preferences in carabids using 64 or 28 species of herbaceous seed. Honek et al. (2003, 2007, 2011) demonstrated that the preference for seeds correlates with carabid body size: on average, smaller species prefer smaller seeds, and *vice versa*. Larger carabids also consume a greater variety of seed species and Harpalini are less specialised than Zabrini (Honek et al. 2007). However, there are other characters such as seed shape, thickness of the testa (Lundgren and Rosentrater 2007) and nutrient content of the seed that affect preference. Similarly to other seed-cracking organisms (e.g. Diaz 1994), mandible size and shape determine the seed preferences of *Notiobia* species occupying fruit fall sites in tropical forests (Arndt and Kirmse 2002; Paarmann et al. 2006), and these preferences are consistent throughout the season (Honek et al. 2006).

Taxonomic affiliation constrains the preferences for food in many insect groups. Earlier research as well as direct field observations have indicated that species of certain genera had specific affinities with respect to their seed preferences. For example, Brandmayr and Zetto Brandmayr (1987) and Zetto Brandmayr (1990) suggested that most *Ditomina* and *Ophonus* (both Harpalini) are associated with Apiaceae, while *Harpalus* (Harpalini) is unspecialised in this sense (Zetto Brandmayr 1990). Hurka (1996) reported that species of the subgenus *Zezea* (Zabrini: *Amara*) may be associated with Poaceae. The existence of a taxonomic constraint has been experimentally confirmed by Honek et al. (2007), who carried out a cafeteria experiment that included 28 seed species and 30 carabid species. They demonstrated that species of Zabrini mostly prefer seeds of *Taraxacum*, while species of Harpalini prefer seeds of *Cirsium* and Viola. Carabids not only distinguished seeds from different families, but they were also able to discriminate between seeds at a finer taxonomic scale, i.e. seeds of different sections of the *Taraxacum officinale* species complex (Honek et al. 2011). The origin of seeds plays a role in some carabid species. For example, Honek et al. (2011) fed Czech carabids with Italian and Czech seeds of the same plant species and found that the beetles preferred the latter. It is likely that the existence of specialisation on particular seeds reduces the competition for food and allows the coexistence of species in the same habitat.

Seeds are nutritious, but their value as food for carabids has not been appropriately recognised until recently. The value of food is best defined by its contribution to the fitness of the consumer (Toft and Bilde 2002). Fitness parameters that are commonly used as criteria for the evaluation of food quality are female fecundity, survival and duration of larval development, and the attainable body size. Zetto Brandmayr (1976) showed better survival in larvae of several species of the genus *Ophonus* when provided with seeds of Apiaceae compared to other seeds or insects. Although Jørgensen and Toft (1997a, b) stimulated further research on this topic (mainly in Europe and Japan), information on how seed diet affects fitness is only available for a small number of species. Adaptations to granivory have evolved to varying degrees in different taxa, and even closely related species may show different strategies (for *Amara*, subgenus *Amara*, compare e.g. Jørgensen and Toft 1997b; Saska and Jarošík^ 2001^; Hurka and Jarošík 2003; Fawki and Toft 2005; Saska 2008; for *Amara*, subgenus *Curtonotus*, compare e.g. Saska 2005; Sasakawa 2007; 2009); for *Notiobia*, see Arndt et al. 1996; Paarmann et al. 2001; Arndt and Kirmse 2002). More interestingly, particular seed diets may have contrasting effects on different fitness traits (Fawki and Toft 2005). The effects of maternal diet (Saskawa 2009) or diet of the previous generations (Hurka and Jarošík 2003) on larval performance are poorly studied. Also, worthy of mention here is the scoring system of Paarmann (2002) used to evaluate larval performance under different dietary regimes. In general, larvae are more specific in their food preferences than adults (Thiele 1977) because of increased selection pressures on larvae (Sasakawa 2007) and due to morphological constraints on the suitability of the available food during the early stages of development (Paarmann et al. 2006). Klimeš and Saska (2010) argued that this selection pressure is highest in the first instar larva and decreases in older instars, with increasing the head width/seed size ratio in larvae and widening the range of edible food items.

**Ant feeding**

Ants are the most abundant group of organisms on Earth in terms of biomass (Hölldobler and Wilson 1990). Not surprisingly they represent an important food source for many other taxa, including carabid beetles. Polyphagous carabid species frequently prey on ants (Thiele 1977; Hengeveld 1980b), and several clades have adapted to ant feeding with some having evolved the highest degree of specialisation, i.e. myrmecophily. In general, biological information is very limited and needs systematic study.

Species that have adapted to feeding on ants have evolved interesting behavioural and morphological adaptations, including chemical mimicry that reduces the risk of being attacked by their hosts (Zetto Brandmayr et al. 2000a; Dinter et al. 2002). Larvae of *Sphallomorpha* (Pseudomorphini) form burrows close to ant nests and attack ants that pass by (Moore 1974). Associations with ants and termites seem to be a joint character for the entire tribe of Pseudomorphini, though evidence is limited (Baehr 1994). Species of the Siagonini also prey on ants ( 1998a, [Bibr B609]). Species of the genus *Siagona* inhabit crevices in the soil near ant nests and attack ants both as adults and larvae, but do not seem to enter ant nests frequently (Bauer et al. 2005). The larvae of some Ozaeini use so-called terminal disks (modified last abdominal segments) for attracting and capturing ants (Di Giulio and Vigna Taglianti 2001; Moore and Di Giulio 2006).

Adults of the North African Anthiini and Graphipterini are free-living but larvae enter ant nests where they prey upon ants to complete their development (Paarmann 1985; Paarmann et al. 1986). The larva of *Thermophilum* (Anthiini) moves freely in the nest after it gains chemical mimicry from ants it has previously attacked (Dinter et al. 2002), and consumes both ants and ant brood (Paarmann and Erbeling 1986). In contrast, the larva of *Graphipterus serrator* forms a chamber inside the ant nest where it stores ant brood before consumption, and hides against ant attacks (Dinter et al. 2002). Species of *Thermophilum*, as well as *Graphipterus serrator*, show preferences for particular ant species, *Graphipterus* being the least selective (Dinter et al. 2002).

True myrmecophily (and perhaps termitophily) evolved in the tribe Paussini, in which morphological and behavioural adaptations are prominent in both adults and larvae (Nagel 1979b; Di Giulio and Moore 2004; Moore and Di Giulio 2006). Although this association is well known, data on food requirements or trophic associations are known for a limited number of taxa only, and this requires further investigation.

**Unique life history strategies – ectoparasitism and the predation of amphibians**

The variety of life history strategies in carabid beetles includes ectoparasitoidism, a strategy otherwise rare in beetles. Parasitoids are insects whose larvae develop at the expense of a single prey individual (a host), which ultimately dies as a result of parasitoid feeding (Vinson 1976). Ectoparasitoid larvae attach to the host body and feed externally on it, while their adults are free-living (Vinson 1976).

Ectoparasitoidism has been described from four carabid genera: *Brachinus* (Brachinini), *Pelecium* (Peleciini), *Lebia* and *Lebistina* (both Lebiini) (Weber et al. 2008), but several related genera show tendencies towards parasitoidism (Erwin 1979a; Frank et al. 2009). The life cycle of a typical carabid ectoparasitoid includes (i) a female depositing eggs in the host habitat when hosts are present; (ii) mobile early instar larva searching for and attaching to a suitable host; (iii) after attachment, a short physogastric feeding phase, typically with rapid ingestion; and (iv) a distinct pre-pupal “resting” phase during which the host is consumed.

Despite the early discovery of ectoparasitoidism in Carabidae (e.g. Wickham 1893; Silvestri 1904), known host associations are few. With one known exception, beetle pupae are the hosts. Larvae of *Lebia* (five species known to be parasitoids) and *Lebistina* (one species) parasitise leaf beetle (Chrysomelidae) pupae (Weber et al. 2008). Larvae of a single undetermined species of *Pelecium* have been observed developing on chrysomelid pupae and millipedes (Salt 1928). Nearctic wetland species of *Brachinus* (seven species) parasitise the pupae of water beetles (Dytiscidae, Gyrinidae, Hydrophilidae) (Saska and Honek^ 2004^). Despite suggestions proposed by Jeannel (1942), the discovery of the hosts for dryland species of *Brachinus* from Europe was only made 60 years later. Saska and Honek( (2004, 2005) successfully reared two species (*Brachinus explodens* and *Brachinus crepitans*) on the pupae of another carabid genus, *Amara*, a finding that has recently been confirmed for *Brachinus elegans* by Makarov and Bokhovko (2005).

Besides direct observations, host-parasite associations have frequently been suggested simply on the basis of co-occurrence of the carabid parasitoid and potential host species. In some cases, however, these observations have led to erroneous predictions (Jeannel 1942; Perez-Zaballos 1985), subsequently refuted because the life cycles of the two suggested partners are not synchronous. Such synchrony has so far been demonstrated only for *Brachinus explodens* and *Brachinus crepitans* (Saska and Honek 2008). Thus, when looking for hosts of *Mastax* or *Aptinus* (both Brachinini) or wetland Palaearctic *Brachinus* species, both co-occurrence and synchrony should be taken into account. More discoveries are probably to be made in the tropics, as that climatic zone contains a vast diversity of lebiine carabids (Ober and Maddison 2008). Research is also needed on the ecology of ectoparasitic carabids to determine the adaptive significance of life history traits of this peculiar strategy. In most cases, available information relates to a brief description of development; only a few species have been studied in detail (Erwin 1967; Juliano 1985; Saska and Honek^ 2004^, 2005, 2008; Weber et al. 2006). Host selection, food utilisation or the adaptive significance of variation in the number of instars (2–5 instead of the typical 3) could produce interesting results. Mimetic complexes have been described between adults of *Lebia* and chrysomelids, including species for which parasitoidism is unknown (Hemenway and Whitcomb 1967), suggesting further trophic associations between the two groups. Focusing on taxa representing transitional evolutionary steps to parasitoidism (Erwin 1979a; Frank et al. 2009) may shed light on the evolution of parasitoidism in Carabidae and in Coleoptera in general.

Carabid beetle larval and adult predation on amphibians has recently been described in Israel. Elron et al. (2007) have shown that larvae of the carabid *Epomis dejeani* preyed upon two amphibian species (*Bufo viridis* and *Hyla savignyi*), confirming an earlier brief note by Moore (1971) from Australia. Subsequently, Wizen and Gasith (2011) performed laboratory experiments, and showed that adults of the two sympatric *Epomis* species in Israel, *Epomis dejeani* and *Epomis circumscriptus*, prey upon five and four amphibian species, respectively. Wizen and Gasith (2011) argue that little is known about the feeding habits of sympatric congeneric insects, and that the partial food overlap of these *Epomis* species warrants further investigation.

### 3.3. Dispersal

Carabid beetles found a rich niche in ecological research through the peculiarities of their dispersal power. Sahlberg (1868) recognised that carabid species exhibit a variety of wing attributes, including wing dimorphism, and that this has implications for their powers of dispersal. Darwin was probably the first to consider the evolutionary and ecological implications of wing polymorphism in Coleoptera after recording high proportions of flightless beetles on the island of Madeira. He hypothesized that flight ability might be evolutionarily disadvantageous for species from insular populations, as they would be more likely to get carried away from the island (Darwin 1859). A few decades later, Darlington turned his attention to the low proportions of macropterous carabids in isolated locations such as islands and mountain tops, and concluded that wing reduction must confer enhanced viability (Darlington 1936, 1943). Lindroth (1988, 1992a, b) studied the wing morphology of carabid assemblages from islands in the Baltic Sea in comparison to control assemblages from nearby mainland sites. He found that the proportions of brachypterous and macropterous species were both lower in insular than in mainland assemblages, even whilst macropterous species were predominant in all of the studied assemblages (see also Ås 1984, Kotze et al. 2000). Dimorphic species, on the other hand, were more numerous in insular than in mainland faunas (Lindroth 1988, 1992a, b). These observations were of fundamental importance to Lindroth’s epic zoogeographical studies, published posthumously in 1992 (Lindroth 1988, 1992a, b). After determining the frequencies of the different wing morphologies in populations of wing-dimorphic carabid species across the Fennoscandian region, Lindroth was able to estimate the relative ages of these populations. On that basis, he theorised about the routes of post-glacial colonisation of Fennoscandia by different species. He was subsequently able to divide the fauna into three elements: Wűrm hibernators, immigrants from a southern route to the west of the Baltic Sea and immigrants from the east. Both Lindroth (1988, 1992a, b) and Den Boer (1970) came to the conclusion that macropterous specimens dominate in recently established populations of dimorphic species, which gradually shift to an increasing proportion of brachypterous individuals as these populations grow older. Observations of pioneering populations of the invasive species *Pterostichus melanarius* in Canada support this model (Niemelä and Spence 1991).

Largely thanks to the work of Piet den Boer and colleagues, subsequent to the Dutch land reclamation projects of the late 1950s, research interest in the dispersal of carabid beetles flourished, and this provided the theme for the first meeting of European carabidologists in Wijster in 1969, which Piet den Boer hosted (see above). Dispersal power was also the theme of the subsequently published proceedings volume, edited by Den Boer (1971, see also Table 2).

Lindroth was keen to determine the genetic mechanism behind wing dimorphism and conducted breeding experiments with the wing-dimorphic species *Pterostichus anthracinus* (Lindroth 1988, 1992a, b). The results he obtained, supported by similar results from studies of other coleopteran taxa, led him to conclude that wing dimorphism is inherited in a simple Mendelian pattern, in which brachyptery is dominant. The late Konjev Desender, in whose honour the 14th ECM was held, performed similar breeding experiments using the wing polymorphic species *Pogonus chalceus*. In this species, crosses between macropterous and brachypterous adults produced offspring with intermediate wing length, suggesting that the genetic control of wing length in this species is polygenic (Desender 1989a). Desender also conducted an exhaustive biometric study of wing development in 300 carabid species indigenous to Belgium and demonstrated that, in addition to brachypterous individuals, also a large proportion of macropterous individuals do not possess functioning flight muscles and are therefore incapable of flight. In the wing-polymorphic *Pterostichus vernalis*, for instance, some populations are entirely macropterous, with functional flight (but see below) muscles even in relatively short-winged individuals, whereas in some other populations even macropterous individuals lack functional flight muscles (Desender 1989b, see also Nelemans 1987). Desender also studied wing morphology in the genus *Calosoma* after research trips to Easter Island and the Galapagos archipelago. Three endemic species appeared to be brachypterous, whereas the supposedly introduced species, *Calosoma granatense,* appeared to be wing polymorphic (Desender et al. 2000).

Berend Aukema conducted breeding experiments with the *Calathus melanocephalus* group to shed further light on the inheritance of dispersal characteristics. Aukema (1990) demonstrated that these species show a simple Mendelian pattern of inheritance of wing morphology, as described by Lindroth, i.e. simple inheritance with brachyptery dominant over macroptery for the two wing dimorphic species *Calathus cinctus* and *Calathus melanocephalus*. However, he also demonstrated that certain environmental factors, such as temperature and food supply, influence expression, with higher temperatures and better food availability resulting in both greater proportion of macropterous individuals (Aukema 1990), and the development of flight muscles (Nelemans 1987). Moreover, long-winged females of these two species had greater fecundity than short-winged females, both in terms of quantity of egg production and duration of egg production (Aukema 1991). This result was somewhat counterintuitive, as a number of other studies of wing dimorphic insects, e.g. Roff (1986) found that brachypterous females are generally more fecund, suggesting that the advantage conferred by brachyptery is enhanced fecundity for females. Furthermore, macropterous females of *Pogonus chalceus* have greater fecundity, suggesting that long wings and functional flight muscles are associated with large body size (Desender 1989b; Aukema 1991).

Work from other invertebrate taxa has suggested that there is a cost in terms of reproductive capacity for flight, with some macropterous females lysing their flight muscles and shedding their wings prior to reproduction, resulting in enhanced reproductive capacity. Among carabids, *Amara plebeja* autolyses its wings and can subsequently regenerate them to facilitate migration between breeding and over-wintering habitats (van Huizen 1977, 1979). This is supported by Matalin’s (1994) observation that reproductive females from window traps invariably have fewer ova than those from pitfall traps. Matalin (1994) also concluded that the choice between flying and walking varies considerably between species and with different stages in the life cycle, with flight activity being favoured by dispersive young adults, shortly after emergence and, in *Harpalus rufipes* and *Harpalus calceatus*, by mature males. Mature adults exhibit the highest walking activity during the breeding season, apparently being the favoured form of locomotion when seeking a mate (Matalin 1994).

Wing morphology alone is not sufficient to describe dispersal ability in carabids. Desender (2000) and Matalin (2003) studied the phenology of carabids in relation to flight muscle development. Desender (2000) investigated the trade-off between dispersal and reproduction in female carabids from the Belgian fauna, and most of the species he studied supported the oogenesis-flight syndrome, i.e. females with ripe ovaries tend not to possess functional flight musculature. This phenomenon was most pronounced for species that reproduce in late summer or autumn and emerge in late spring (Desender 2000). Matalin (2003) concluded that in females of large species, wing muscles decline during a period of increasing body mass, after development of the gonads.

In addition to the wealth of material on dispersal by flight, carabidologists have also investigated running activity, demonstrating that larger *Carabus* species run slower than smaller carabids, though in Pterostichinae and Harpalinae, larger species are faster (Mossakowski and Stier 1983). Temperature has a significant effect on running activity in *Carabus auronitens* (Althoff et al. 1994). Clearly the expression of dispersal ability in carabid beetles is highly complex, being governed by environmental and life cycle factors, in addition to genetic control. It is equally clear that there are still many unresolved issues regarding the dispersal of carabids and we are likely to see studies on this topic at future ECMs. In particular, ongoing land-use change and habitat fragmentation, exacerbated by the influence of climate change, mean stronger selective advantages for species with better powers of dispersal. A major challenge for the scientific community will be to discern evolutionary changes in response to this selective pressure. In conservation, the main challenge will be to develop strategies for the conservation of species with poor powers of dispersal.

## 4 Methods

### 4.1. Methodological approaches

Methods influence the way we approach, perceive, and understand the world. All methods have strengths and weaknesses, which make certain things to be easily noticed while others remain hidden or un-emphasised – and such effects of the methods on knowledge often go unnoticed or are unappreciated by researchers. Carabid research has long been dominated by observation and description, but there still remains much to be observed and described about carabids. However, the prevalence of certain methods in carabid research (e.g. pitfall trapping as a collection method, see below) has put a strong stamp on the amount and structure of our knowledge about carabids. Some of the resulting biases are mentioned below; this list is illustrative, not exhaustive.

*Prevalence of knowledge about adults:* Due to the epigaeic activity of the adults, and the fact that they are more easily collected, manipulated and kept in the laboratory, there is an overwhelming disparity about our knowledge on the ecology of the different life stages of carabid beetles. Our knowledge on carabids was (Lövei and Sunderland 1996) and remains primarily determined by knowledge about adults. A search on Web of Science with the term “carabid* OR ground beetl*” between 2000–2009 yielded 3186 papers, only 460 remaining when this was combined with the term “larv*” (search made by G Lövei, on 4 February 2011).

*Geographical unevenness in the origin of our knowledge:* This is a general phenomenon: we know that the tropics is more species rich, in general, than the temperate region (already mentioned by Darwin 1859), yet most of our research effort is still directed towards temperate ground beetles. Of the above computer search on ground beetles, only 80 of the original 3186 papers remained when the additional term “tropic*” was introduced. We can safely predict important new understanding emerging from more detailed studies performed in more southerly regions; many of the techniques formerly restricted to developed countries can now be usefully employed in more tropical areas.

*Biased perception of carabids as predators:* Predators and predation keep us fascinated, possibly because early humans have been both hunters and hunted. However, this colours our perception of the world (see *Carabid beetle food* above). In the case of carabids, the fact that many species will attack prey offered to them, especially in the laboratory, and that many beetles are indeed fast-moving predators, has led to a widely-held belief that carabids are predators. Carabidologists (mostly) know better, but we have been a bit lax to actively dispel this notion among ecologists, natural historians, and the general public. In relatively recent literature, one still comes across this perception (Braun et al. 2004), and in some cases, elaborate theories are built on such shaky grounds (Lövei and Magura 2006).

*The rarity of testable hypotheses*: Due to a history of descriptive studies, there seems to be a general rarity of precisely formulated, testable hypotheses. Many studies have the only justification that “we do not yet know, so let’s find out”. With increasingly fierce competition for funding and publication, such arguments do not carry much weight. An additional advantage of formulating hypotheses is that it forces us to think ahead: what is to be expected? Why? However, hypotheses should be well formulated (see Ford 2009; Underwood 2009). In the literature (not only in carabidology) one often encounters the “null hypothesis” formulated as “we expect no differences will be found”. Do researchers really expect that “nothing will happen”? If so, why is the experiment worth performing? Indeed, in the real world the null hypothesis is rarely if ever true as there will always be differences between effects. What is of importance is the magnitude, i.e. effect size, and precision, i.e. confidence interval of the effect (Nakagawa and Cuthill 2007; Läärä 2009). The careful separation of hypothesis formulation vs. the Popperian way of arriving at scientific evidence should not be confused – but often is.

The overall task is unchanged: to understand what made carabids such an evolutionarily successful group. In order to answer this question, one has to quantitatively continue to document the patterns of occurrence of members of this group – this is a logistical, not a methodological challenge. Among the promising “methodological approaches”, modern population genetical toolkits are well used, with several interesting results – it would be good to take these and use them in extra-European habitats as well. Gene expression study methods have recently developed and simplified considerably (Ouborg and Vriezen 2007), and facilitate the study of some interesting ecological questions, such as reaction to such factors as stress and food selection. Modern methods, such as those of ecological immunity, also allow a more refined characterisation of ground beetle reactions to habitat quality.

### 4.2. Analysing pitfall-trapped carabid data

**Figure 2.**
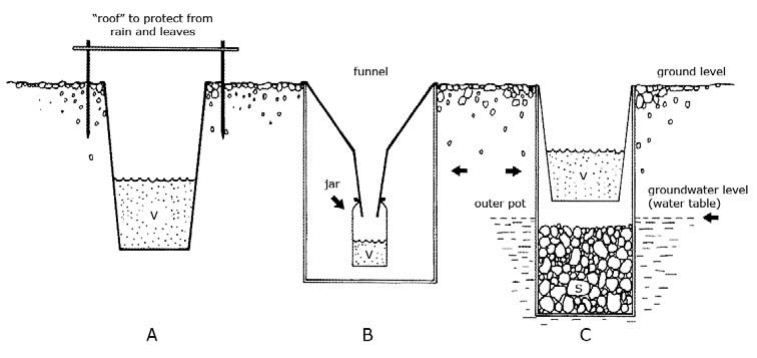
Different pitfall types. **A** = Jar or yoghurt can. **B** and **C** = traps with an outer can to make collecting of the sample easier. **B** = funnel trap with small jar. **C** = trap for moist biotopes (the outer can contains gravel or stones to prevent the can from being pushed up by groundwater). **V** = preservative (usually formaldehyde 3–4% or propylene glycol), **S** = stones or gravel.

Pitfall trapping is the best-known collection method used by carabidologists, especially in ecological studies (Lövei and Sunderland 1996). The method, originally described nearly 80 years ago (Barber 1931) and later often referred to as Barber traps (Thiele 1977), is cheap, easy to use and once set up, operates by itself. It allows for adequate replication in field-based studies, and collects large samples (see Fig. 2 for examples of a few commonly used pitfall traps).

One of the most convenient features of pitfall trapping is also its main disadvantage, because the resulting catch, although beguilingly countable, is not a measure of density, but of activity density. Carabidologists have recognised this and other drawbacks of pitfall trapping, which have often been discussed in the literature from Greenslade (1964), Thiele (1977) and Lövei and Sunderland (1996) to Holland (2002) and regularly at ECMs. However, the method has not been subject to rigorous, thorough testing, nor to a systematic review, and consequently, most carabidologists tip their hat at the problem, then proceed to ignore it, and often use sophisticated evaluation methods to answer important research questions. Needless to say that if these drawbacks in pitfall-trapped samples remain unresolved, this brings into question any analysis using assemblage data, such as ordination techniques, diversity indices, the determination of dominance structure and any ecological analysis or testing of theory.

While the sharpening of research questions before starting trapping is a salutary piece of advice, which will also influence the type and arrangement of traps, some problems associated with pitfall traps for general carabid beetle studies have reached a general consensus. Several of the aspects below are, however, still ignored but could be easily fixed. These include that (i) an odourless preservative is preferred, because formalin, for example, seems to attract some species and repel others (Thiele 1977); (ii) the traps should have a cover to prevent flooding, desiccation, scavenging and bycatch – a funnel to prevent escape and reduce bycatch also helps (Lange et al. 2011); (iii) traps should preferably not be used solitarily, but placed in series of at least three to five traps at distances of less than 10 m apart in order to optimise the catch and to overcome occasional trap losses; (iv) distances between sampling plots (single traps or trap groups) should be large enough to allow for sample independence (this distance will, of course, depend on the dispersal power of the focal species, see e.g. Digweed et al. 1995); and (v) the question of missing samples that inevitably occur when large numbers of traps are used over long time periods (see below). Important challenges that await study and resolution are: (i) that trap numbers and length of the trapping period do not contribute equally to the catch (Lövei and Magura 2011); (ii) how to reliably minimise the impact of trapping on assemblages and protected species (the methods of partial seasonal samples and pulsating samples, for example, have been suggested: Sapia et al. 2005); and (iii) the challenge of non-destructive carabid sampling (Bowie and Frampton 2004), such as radiotelemetry (see Negro et al. 2008).

**Figure 3.**
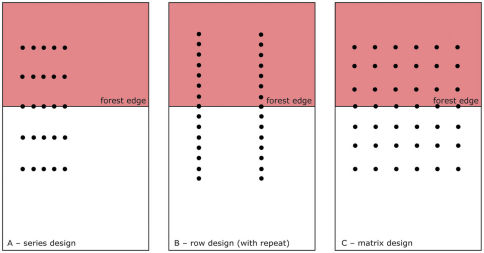
Examples of pitfall trap placements across a forest edge.

The arrangement of pitfall traps in the field depends on the research question asked. The most popular research questions include: (i) *Faunistic investigations* intended to obtain an accurate species list of a given area. Here many pitfall traps should be used, also along gradients and at biotope edges; it seems that the spatial aspect is more important than the temporal one, i.e. it is better to have many traps for shorter periods of time than fewer traps for longer time periods (Lövei and Magura 2011). (ii) *Community or gradient studies* intended to investigate the (typical) fauna of different biotopes or at different positions along a gradient. In this case series of traps per biotope or gradient position can be used (Fig. 3a) with independent replicates (with sufficient distances between the series, see above). An example of this is the Globenet project (Niemelä et al. 2002). In some cases a row design with repeats will generate more precise information (Fig. 3b), especially when short-term movements of species along gradients are expected. The same holds for different treatments in an experimental design, such as (iii) *Biological studies* investigating e.g. the periodicity of one or more species within a year, to be eventually compared with different biotopes or years in phenological and/or climate studies (e.g. do species reproduce earlier or later during warmer periods or in different biotopes?). In the case of (iv) *Biological studies* investigating diurnal rhythms or movements of adults and larvae, a grid or matrix design (Fig. 3c) is recommended; and (v) *Population studies* intended to investigate the response of populations to biotic and abiotic environmental factors. Here, estimates of population densities are required and, as such, pitfall-trapped data need to be interpreted with caution.

The fact that pitfall catches are a function of the species’ true population size and its activity (activity-density: Greenslade 1964; Thomas et al. 1998), creates specific statistical problems. Continuous sampling over the whole activity period can cause a potentially serious problem when the catch is analysed. Trap losses can occur at any time during the activity period and have traditionally been dealt with by standardising the catch to 100 trapping days without taking into account variability in activity across the season (Kotze and Niemelä 2002; Niemelä et al. 2002). For example, some species are more active in the spring or autumn (see *Life history strategies and rhythms* above), while others are active throughout the summer months. As such, a trap lost at the beginning of the continuous sampling period will have a different effect on the estimated activity-density of a spring-active species, for example, than if the trap is lost at a later stage when activity is low. When the research question involves study of the response of separate species to an environmental gradient, statistical models in which seasonality (or visit) is added as a free factor and sampling effort (number of trapping days per visit) as an offset term, and in which the response variable is specified as following a negative binomial distribution, seem to correct for seasonality and trap losses appropriately.

The reason for specifying activity-density (or abundance) data as following a negative binomial distribution (and not a Gaussian distribution, as is often done) is that ecological field data (here counts of individuals or species) seldom follow the assumptions of classical parametric statistics (Dalthorp 2004). Carabid beetles (both in terms of abundance and species) are often aggregated in space (Niemelä et al. 1986, 1992; Thomas et al. 1998) and sampling them is likely to produce an expected variance that is greater than the expected mean. Such ‘clumped’ counts data appear to be most appropriately analysed by models that incorporate extra variation, such as the negative binomial distribution (see White and Bennetts 1996; Dalthorp 2004), or quasi-Poisson methods (Ver Hoef and Boveng 2007; e.g. Elek et al. 2010). Another important advantage of using methods designed for dealing with count data (negative binomial, Poisson) is that the response variable (number of individuals or species) does not need to be transformed to comply with the assumptions of parametric test statistics, such as analysis of variance, t-test or linear regression. Surprisingly, abundance and species richness data are often log-transformed for subsequent use in parametric test procedures, even though textbooks on statistical methods in ecology (Sokal and Rohlf 1995; Crawley 2003) recommend the use of the square-root transformation to normalise count data. Nevertheless, neither square root nor log-transformed count data (for use in parametric tests) performed as well as *non*-transformed data (for use in a negative binomial model) (O’Hara and Kotze 2010). A possible reason for this is that count data often contain many zero values, which have to be fudged (when a log transformation is performed) by adding 0.1 or 1 to every observation – which may have unforeseen effects on estimates.

Another problem occurs when the activity density results for different species are compared. Since each species reacts differently to pitfall traps, their “catchability” will also differ, subsequently with more or less incomparable results between species. A possible solution, suggested by Den Boer, is to standardise the catches per species over the sampling sites (Turin et al. 1991). After standardisation, with the obtained “relative abundances”, multivariate methods (calculating (dis)similarities, clustering and ordination) can be used to analyse the data. Similar classifications have been carried out for Britain (Luff et al. 1989; Eyre and Luff, 1990; Mccracken 1994; Anderson et al. 2000). Although the approach of correcting and standardising the data was quite different from the Dutch method, the results for classification of the carabid habitats in the Netherlands and Britain were very similar. A study of the carabid fauna of Trento, Italy (Bonavita and Chemini 1996) in a deviating trans-alpine fauna, revealed highly corresponding results for the classification of the 48 (out of 57) species common to Italy and northern Europe. A relatively simple and flexible method developed by Dufrêne and Legendre (1997) to classify a Belgian dataset (the IndVal procedure), has the advantage that it is insensitive to the relative abundances of species. We contend that the problems associated with the comparison of assemblages sampled by pitfall trapping are still not fully resolved, but the above confirm that this method has merit in many types of investigations.

## 5 Population dynamics and long-term research

Since the 1960s the population dynamics of carabid beetles has been subject to the study of population persistence. During this time, discussion has revolved around how the size of populations and their fluctuations have been established, resulting in two popular theories. The first theory postulates that population sizes are balanced within narrow limits by density dependent processes, a feedback mechanism in which predators, parasites, competitors for food and other biotic aspects of the environment are involved, resulting in the regulation of population size (see Nicholson 1958). The second theory argues that the founding and re-founding of local populations take place, driven by dispersal, small population size and extinction, heterogeneity of the environment, the distinction between local (sub) and natural (entire) populations, and the genetic plasticity of species in relation to different components of the environment and to fluctuations of population size (Andrewartha and Birch 1954). Den Boer tested the latter theory by using carabid beetles as a model group. In 1959 he started pitfall trapping at several locations in the Dwingelderveld, a large area of heathland in the Netherlands, which he regarded as home to large natural populations of several carabid species. This founding/re-founding theory, the concept of metapopulation, states that natural populations consist of many local populations or colonies. Indeed, Den Boer was able to show that in a large area many local populations or interacting groups of carabids fluctuated in numbers of individuals in space and time. From these results the “spreading of risk” theory was derived (Den Boer 1968, see *European Carabidologists’ Meetings (ECMs)* above).

The significance of dispersal in founding, re-founding and establishment of populations was confirmed during the first ECM (see Introduction). However, the role of density dependent processes was not resolved. In 1970 in Oosterbeek, the Netherlands, an entire symposium on the *Dynamics of Populations* (Den Boer and Gradwell 1971) was devoted to whether or not populations were regulated. Some contributors showed examples in which density-dependent processes seemed to govern the abundance of a species, whereas others showed the opposite, so the discussion continued. Later on, again using carabid beetles, several studies were conducted to test the density dependence hypothesis. For instance Baars and Van Dijk (1984) were able to show that the number of eggs in the ovaries of females was negatively correlated with the mean density around pitfall traps. However, later on Van Dijk and Den Boer (1992) demonstrated that egg and larval mortality were too high to compensate for egg production. It was concluded that the density dependent relationship could hardly play an important role in the dynamics of the populations of *Calathus melanocephalus*, as shown by Baars and Van Dijk (1984). In *Pterostichus oblongopunctatus* the amount of food available affects the number of eggs laid. Heessen (1981) suggested that this would regulate population dynamics of this species. However, Szyszko (1981) and Den Boer (1986) observed that population explosions of certain prey species lead to a strong decline in some carabids. Vermeulen and Szyszko (1992) were able to show that in order to maintain a high level of egg production, *Pterostichus oblongopunctatus* has to switch prey. Presumably the right mixture of amino acids and the quality of nitrogen (White 1993) are essential for a high level of egg production. Another study on regulation in carabid populations was carried out by Brunsting et al. (1986). They showed that cannibalism occurs between larvae of *Pterostichus oblongopunctatus* and suggested that this phenomenon would regulate population size. However, Vermeulen (1986) could not find differences in raising *Pterostichus oblongopunctatus* under circumstances in which cannibalism was included and excluded, suggesting that larvae may not be actively searching for other larvae of the same species to feed upon. Cannibalism might take place only under extremely high, unnatural densities. Also, the role of competition in the population dynamics of carabid beetles has not been convincingly demonstrated so far. For example, Loreau (1990) found only weak evidence for competitive regulation in *Abax ater* populations. He suggested that competition might only be significant in dominant species. On the other hand, (Den Boer (1980, 1985) and Niemelä (1993) showed that competition hardly plays any role in determining population size. The discussion on whether or not regulation plays an important role in population dynamics led to a second symposium on population dynamics, this time held in Poland in 1992 (Den Boer et al. 1993). However, again only a discussion for and against regulation resulted. After this meeting the subject quickly went out of fashion and was not discussed in this way again. In 1996, Den Boer and Reddingius wrote a book in which they reviewed all the population dynamic theories so far.

At present, it is generally accepted that the persistence of carabid populations depends on the availability of sufficient suitable habitat over long periods of time, as well as on habitat quality. The latter was nicely illustrated in the Dwingelderveld, the Netherlands. These heathlands have nitrified from the 1970s onwards, associated with an almost complete disappearance of *Carabus nitens* there. A few years after the removal of the nutrient-rich topsoil layer by sod cutting, however, this species was again recorded in high numbers (Van Essen 1993). A similar recovery is now seen in the Mantingerveld, the Netherlands, for the same species since 2007 (Rikjan Vermeulen, pers. obs.). Because of the turnover in local populations, dispersal is necessary for a given species to (re)colonise areas where habitat patches are small. The classical technique for investigating these processes has been mark and recapture. Using modern techniques, e.g. simulation programmes (Persigehl et al. 2004) and genetic techniques (e.g. Drees et al. 2011), the relationships between populations can be demonstrated more easily.

Permanently set-up pitfall traps give an impression of the activity of different species during different seasons and between years, and produce relative estimates of population fluctuations for a particular species. In 1959, several series of permanent pitfall traps were initiated in the Dwingelderveld and later, in 1963, in the Mantingerveld (Den Boer and Van Dijk 1994). Carabid beetles from these series were collected on a weekly basis. Results from the first 6–7 years showed considerable fluctuations in the total number of individuals of a particular species collected per series between successive years. This fluctuating pattern was also different between each separate catching series within an area in the same year. These observations of asynchronous fluctuations in catches of a particular species were instrumental in the development of the “spreading of risk” theory by Piet den Boer (see above). Environmental conditions since the establishment of these series also changed. At the end of the 1960s the ground-water table gradually receded and during the 1970s the effects of air pollution became apparent: increasing acidification and eutrophication of the upper soil layers and the subsequent replacement of both *Calluna* and *Erica* by grasses. At the end of the 1980s, the local nature management authority started to artificially raise the water table, which subsequently reached its pre-1960s level during 2010–2011. At the same time the grassy vegetation, together with the polluted top soil layer, was removed by sod cutting, and grazing by cows and sheep has subsequently been introduced. Moreover, the average temperature of the area had increased by 1 oC in the last few decades. Both the increase in temperature and the hours of sunshine appear to be significant from 1988 onwards (Prins et al. 2007).

Since the establishment of these series of pitfall traps, the composition of the carabid beetle fauna has changed continuously. In the beginning of the 1970s species such as *Agonum krynickii*, *Carabus cancellatus*, *Cicindela sylvatica* and *Cicindela germanica* disappeared completely from the catches, followed by *Amara quenseli* and *Amara praetermissa*. During the same period, species such as *Carabus nitens*, *Harpalus solitaris* and *Amara infima* decreased significantly in numbers. The climate did not change significantly during this period, and it can be speculated that changes in the environment, as mentioned above, and habitat fragmentation (in the case of Hullenzand, Mantingerveld) may be responsible for these local extinctions and changes in population numbers. From the end of the 1990s, species such as *Agonum ericeti*, *Cymindis vaporariorum* and *Cymindis macularis* disappeared from the catches. This may be a consequence of climate change, since during this period environmental conditions in the heathlands improved. This is well illustrated for *Carabus nitens*, which became rather abundant during this period, as well as for *Carabus arvensis*, *Nebria salina* and *Harpalus solitaris*. From 1990 to^ 2004^, ten species not previously recorded from these areas have been collected (Vermeulen et al. 2004). Recently, two records of *Agonum viridicupreum* can be added to this list (Rikjan Vermeulen pers. obs.). Apart from one, all of these newly recorded species have their center of distribution south of the Netherlands, suggesting that their appearance is related to climate warming. Similarly, the virtual disappearance of the northern species, *Agonum ericeti*, may be related to this phenomenon. Adequate management may, to a limited extent, compensate for the effects of climate change. The northerly distributed *Carabus nitens* that almost disappeared from both the Mantinger- and Dwingelderveld, made a rapid comeback after top-soil removal and sod-cutting.

However, the dramatic decline and extinction of the highly hygrophylic *Carabus clatratus* in Italy may not be entirely related to climate change. *Carabus clatratus* is one of the most localised and endangered carabid species in Europe, and its disappearance from Italy, and possibly also France, is possibly a consequence of the colonisation of its wet biotopes by the alien red swamp crayfish, *Procambarus clarkii*, which preys on adults of *Carabus clatratus* (Casale and Busato 2008).

Long-term data on weekly catches can also be used to monitor phenological changes in species. For example, compared to the period prior to 1988, the activity of *Amara equestris*, *Carabus arvensis*, *Poecilus lepidus* and *Poecilus versicolor* started earlier in the season.

As far as the consequences of climate change, management and other environmental changes are concerned, it is of great importance to continue long-term observational studies of carabid beetles, such as that in Drenthe, so that future changes can be monitored and possibly explained. Such long-term sampling programmes for carabid beetles are also known from Poland, Germany and Italy.

## 6 Bioindicators

Carabids are excellent model organisms for research on ecological and conservation theory. These beetles readily respond to abiotic and biotic variation, and to disturbances and management (e.g. Lövei and Sunderland 1996; Rainio and Niemelä 2003). This evidence has led many to suggest carabids to function as ‘indicators’. An indicator is a taxon or a structure “*whose characteristics* (...) *are used as an index of attributes too difficult, inconvenient, or expensive to measure for other species or environmental conditions of interest*” (Landres et al. 1988). However, using this definition many, if not most, of carabid ‘indicator’ studies appear to only demonstrate individualistic responses to environmental variation. But instead of investing resources for finding new indicator taxa, environmental managers should test and select taxa that are already well known and easily sampled, and that cover multiple dimensions of biodiversity (Taylor and Doran^ 2001^), and critically evaluate their indicator functioning (Langor and Spence 2006). Carabids fulfil the former but the latter aspect requires further attention.

European carabids have certain qualities that make them good candidates for indicators. They are taxonomically well known, with relatively stable systematics, and their ecology has been widely studied (Lövei and Sunderland 1996). Variation in carabid morphology, life history strategies and small-scale abiotic and biotic requirements are extensively documented (e.g. Lindroth 1961–1969, 1985, 1986). Carabids also respond predictably to not only small-scale but also to landscape- and even continent-level phenomena (e.g. Hengeveld 1987; Kotze and O’Hara 2003; Koivula and Spence 2006). Moreover, they are relatively easy to collect in high numbers using standard methods. But can carabids reflect environmental variation in ways useful for conservation assessment purposes? Knowledge of carabid indicator functioning, using the categories listed in Lindenmayer et al. (2000), is briefly summarised below (see Koivula 2011 for a complete evaluation).

**i. Taxon indicators.** The presence of a taxon indicator reflects the presence of a set of other species, and its absence indicates the lack of the entire set of species. Perfect multi-taxon richness overlaps may be rare (e.g. Jonsson and Jonsell^ 1999^; Sætersdal et al. 2005; Similä et al. 2006), which highlights the importance of using multiple taxa in environmental assessments (Taylor and Doran^ 2001^; Duelli and Obrist 2003). Carabid functioning as taxon indicators mostly relies on weak correlations among taxa.

**ii. Keystone indicators.** These species affect their environment disproportionately strongly relative to their abundance. In field and laboratory conditions, carabids forage on slugs and pest insects (e.g. Kromp^ 1999^). Hance (1987) showed that, using enclosures with different carabid densities, carabids have the potential to significantly prey on pest insects foraging on crop plants with economic benefits.

**iii. Pollution indicators.** These taxa reflect human-altered abiotic conditions. Heavy metals in the soil negatively affect carabids (e.g. Maryański et al. 2002; Ermakov 2004), and in agro-ecosystems, pesticides and fertilizers affect carabids, at least in the short term (e.g. Huusela-Veistola 1996; Kromp^ 1999^).

**iv. Dominant indicators.** These taxa make up much of the total biomass or the number of individuals in an area of interest and predict particular ecosystems or assemblages. Many common carabid species are succession and habitat-type generalists (Lindroth 1985, 1986; Niemelä et al. 2007), so their numbers may not indicate aspects useful for conservation or management. Mean Individual Biomass (MIB), on the other hand, links carabid biomass to succession without considering species entities (Szyszko et al. 2000). However, the ‘behaviour’ of MIB along succession should be examined in detail before applying it in conservation and management.

**v. Environmental indicators.** These should reliably reflect particular environmental conditions. Although carabids have the potential to reflect soils, wetness and habitat-type variation (e.g. Thiele 1977; Lindroth 1985, 1986), they cannot currently compete with plants as indicators of these factors.

**vi. Early-warning signallers (true bio-indicators).** These taxa are extremely sensitive to changing environmental conditions. Carabid evidence is scarce, but some carabids have apparently undergone shifts of tens of metres in altitude over 10–20 years (Assmann 2009; Pizzolotto 2009, David Kavanaugh, pers. comm.), coinciding with climate warming (Parry et al. 2007, see *Population dynamics and long-term research* above). These observations suggest good potential in, for example, climate-change and urban-spread research.

**vii. Disturbance indicators.** These taxa reflect natural and human-caused disturbances. Carabids readily respond to agriculture and forestry (for reviews, see Lövei and Sunderland 1996; Kromp^ 1999^; Niemelä et al. 2007). Their indicator functioning may hold at a general level: they respond similarly to environmental change as many other taxa do (e.g. Barbaro et al. 2005). But indicators should not be used for self-evident patterns: the ecological impact of clear-cutting, for example, does not require an indicator.

Clearly, carabids have good potential for becoming useful indicators for conservationists and environmental managers. Certain obstacles still need to be overcome. First, the functioning and accuracy of carabids to predict habitats or species requiring conservation action should be critically evaluated. According to the indicator definition of Landres et al. (1988), none of the above examples indicate that carabids function as particularly useful indicators. Thus, for a conservationist, carabid responses should be considered as individualistic as long as there is no evidence for their responses to reliably predict responses of threatened taxa or particular, difficult-to-observe conditions. This is important because there is very little room for error if threatened species or habitats are at stake. Strict tests must thus be applied to evaluate indicator functioning (Langor and Spence 2006). Second, the relationship between carabid responses and other taxa should be considerably clarified (Rainio and Niemelä 2003) before using these beetles in environmental assessments. Third, it is unclear whether carabids reflect aspects not attainable using other indicators (apart from their individualistic response) and whether conditions exist under which carabids really are the most cost-efficient indicator taxon. Currently widely used, easy-to-use, relatively cheap and economic tools for assessing the state of the environment include vegetation, habitat structural elements, satellite and aerial photos, as well as weather and land-use inventory data.

The focus of carabidologists should perhaps be changed from total species richness to the indicator potential of single species, groups of specialists or functional groups. We lack an explicitly defined ‘niche’ of these beetles in environmental assessment protocols. Cases for carabids fulfilling the conservationists’ definition for a useful indicator (Landres et al. 1988) will possibly be documented in the near future, but their indicator functioning may always remain context specific.

## 7 Carabid conservation, protection and habitat management

Conservation may mean protecting particular species or patches of habitat against alteration, generally human-caused, but the term may also include operations characterised by an active human role (e.g. Freitag and Kavanaugh 1993; Den Boer and Van Dijk 1994; Sutherland 1998; Gaston and Spicer^ 2004^). Examples include the maintenance of areas of high natural value, the restoration of patches to a state they are presumed to once have represented (often referred to as ‘natural’ state), and the artificial conversion of one habitat type to another. The latter may be required in landscapes where habitat for a threatened species has become rare (see Negro et al. 2008) and new habitat patches are unlikely to appear through natural processes. Such cases might be found, for example, within urban areas. These active operations of patch maintenance, restoration and creation are collectively called ‘conservation management’.

Insect conservation management is a relatively new research discipline, both generally and in the context of carabid beetles (e.g. Lewis et al. 2007; Leather et al. 2008; New 2010). The restoration and artificial creation of habitats − two elements of conservation management − have been important components of carabid conservation since the 1980s (e.g. Thomas 1990; Främbs 1990; Blake et al. 1996). Conservation became an important topic for the ECMs since the Hungarian meeting in 1986. Before that meeting, conservation issues were only occasionally discussed, but from then on, both conservation in general (e.g. identifying diversity hotspots and gathering data on endemic and rare species) and practical conservation management in particular have been among key topics and have altogether consistently made up over 20% of papers in the proceedings. Generally, almost any piece of knowledge on carabid ecology can be applied in conservation-management policy and action to support these beetles and associated epigaeic fauna. In Europe and North America, information necessary for efficient conservation − on carabid ecology and threats − is readily available (Maelfait et al. 1994; Lövei and Sunderland 1996; see also national lists of threatened species). However, the functioning of active management for the benefit of threatened carabid species urgently demands critical evaluation and detailed information. For instance, according to Desender et al. (2010), the decline of carabid beetles in Belgium between the period <1950 and 1950–1985, had halted for a considerable number of species. During the period 1986–2008, however, 60% of these species still had not reached the same distribution area as in the first half of the 20th century, notwithstanding many initiatives and large scale active management. Most of these species now only occur in large and high-quality nature reserves with the last remnants of semi-natural biotopes and have, at present, little or no possibilities to further increase their distribution range.

Here the advances in conservation management, mostly as derived from the proceedings of the previous ECMs are discussed under four topics: (i) Which species characteristics are particularly associated with threatened species? (ii) In which habitat types can conservation of carabids best be realised? (iii) What do we know about habitat connectivity as a way to conserve carabids? (iv) How does conservation management of habitats affect carabids?

**i. Ecological and habitat characteristics of threatened species.** To study which ecological and habitat characteristics of carabids are associated with species being threatened, national species lists and their IUCN categories for five countries are used as examples: Belgium, Sweden, Denmark, Norway and Finland (respectively Desender et al. 2008a, b; Gärdenfors 2005; Pedersen and Wind 2009; Kålås et al. 2006; Rassi et al. 2001). This dataset is complemented with four regional lists of threatened species from Niedersachsen and Bremen, Germany; Nordrhein-Westfalen, Germany; Wadden Sea area; and a preliminary red list for Drenthe, the Netherlands (respectively Assmann et al. 2002; Schüle and Terlutter 1998; Mahler et al. 1996; Noordijk and Vermeulen 2009). For analytical purposes, species characteristics were collected from Lindroth (1985, 1986), Desender (1986), Turin and Den Boer (1988), Desender and Turin (1989), Turin (2000), Anonymous (2006) and Desender et al. (2008a). Several characteristics were evaluated, such as the roles of body size, wing morphology, and associations with shadiness and moisture. This evaluation was done by calculating percentages per size, wing morphology, shadiness and moisture classes for all species (for the five countries), for species classified as threatened by IUCN categories NT (Near Threatened), VU (VUlnerable), EN (ENdangered), CR (CRitically endangered) and EW (Extinct in the Wild; also RE, i.e. Regionally Extinct, in some national lists), and also the proportion of threatened species over all species within a given class. Occasionally, certain information for some species was lacking and these were (partly) removed from the analysis. For example, if for a certain species information was unavailable on wing morphology, it was omitted from the wing morphology analysis but retained in other analyses.

Carabids mostly fell into mid-size classes (43–46% of all species were 4.1–8.0 mm and 28–31% were 8.1–16.0 mm), were macropterous (64–71%) and were associated with open areas (63–64%), but were quite evenly distributed among moisture-association classes (see columns “All” in Table 3). Carabids classified as being threatened roughly complied with these figures (columns “IUCN” in Table 3): also these species were mostly mid-sized (26–50% were 4.1–8.0 mm and 24–39% were 8.1–16.0 mm), macropterous (64–81%) and open-area associated (63–79%; very shady habitats had only 2–12%). However, threatened species were more often associated with either very wet (34–53%) or very dry habitats (32–47%) than with “average” or moist/dryish conditions (12–30%). This dichotomous association with both very dry and very wet habitats was much more pronounced in the four Nordic countries and in the two areas in Germany than in Belgium or in Drenthe (Table 3).

**Table 3.** Morphological and habitat-association characteristics of all carabid species found in a given area (“All” columns; % of species), of species classified as threatened according to the IUCN (“IUCN”; categories NT, EN, VU, CR and EW pooled; % of species), and proportion of threatened species of all species within a given category (“% IUCN”). For example, the value “50” for BEL % IUCN >16 mm indicates that in Belgium, of all species with body size >16 mm, 50% are considered threatened. Values for “All“ and “IUCN“ columns make up 100% for each area/country. BEL = Belgium; SWE = Sweden; DEN = Denmark; NOR = Norway; FIN = Finland; Niede = Niedersachsen and Bremen, Germany; Nordr = Nordrhein-Westfalen, Germany; Wadde = Wadden Sea area; and Drent = Drenthe, the Netherlands (proposed Red Data list). For the last four areas, only species classified as threatened according to the IUCN are shown. For species and their characteristics data, see text.

*Classes*	*BEL*			*SWE*			*DEN*			
	*All*	*IUCN*	*% IUCN*	*All*	*IUCN*	*% IUCN*	*All*	*IUCN*	*% IUCN*	
*Body size*										
0.1-4.0 mm	18	13	18	19	16	14	19	9	13	
4.1-8.0 mm	44	40	25	44	31	11	43	38	25	
8.1-16.0 mm	31	35	31	29	40	23	30	41	39	
>16 mm	7	12	50	8	13	28	8	12	46	
*Wing morphology*										
Macropt	71	69	23	68	69	15	66	68	29	
Poly/dimo	16	18	26	19	19	14	14	16	33	
Brachypt	13	13	23	13	12	13	20	16	23	
*Shadiness*										
Shady (forest)	11	8	24	11	11	17	11	10	25	
Generalist	26	23	29	25	11	7	25	26	29	
Open	63	69	36	64	78	20	64	64	28	
*Moisture*										
Water/wet	39	35	29	38	35	15	37	42	32	
Moist-dryish	29	23	26	30	18	10	31	22	20	
Dry	32	42	43	32	47	24	32	36	30	
	*NOR*			*FIN*			*Niede*	*Nordr*	*Wadde*	*Drent*
*Classes*	*All*	*IUCN*	*% IUCN*	*All*	*IUCN*	*% IUCN*	*IUCN*	*IUCN*	*IUCN*	*IUCN*
*Body size*										
0.1-4.0 mm	18	13	13	18	15	10	19	17	19	16
4.1-8.0 mm	46	38	15	45	26	7	44	40	50	33
8.1-16.0 mm	28	36	24	31	53	21	30	34	24	39
>16 mm	8	13	30	6	6	12	7	9	7	12
*Wing morphology*										
Macropt	64	63	16	70	81	12	71	74	74	64
Poly/dimo	21	16	13	17	15	9	17	14	21	21
Brachypt	15	21	23	13	4	3	12	12	5	15
*Shadiness*										
Shady (forest)	10	9	15	10	3	4	6	7	2	12
Generalist	26	21	15	27	21	9	28	30	19	21
Open	64	70	20	63	76	15	66	63	79	67
*Moisture*										
Water/wet	37	47	22	40	47	14	46	51	53	31
Moist-dryish	32	17	10	30	12	5	16	17	14	30
Dry	31	36	20	30	41	17	38	32	33	39

The proportion of threatened species over all species in the five countries revealed some important issues (columns “% IUCN” in Table 3). First of all, relative to the total number of species per category, larger species tended to be more often threatened than smaller species (see Kotze and O’Hara 2003). For size classes 8.1–16.0 mm and >16 mm, the proportions of threatened species were 21–39% and 12–50%, respectively, whereas for the size classes 0.1–4.0 mm and 4.1–8.0 mm, they were 10–18% and 7–25%, respectively. After pooling species into larger (>8.1 mm) and smaller (0.1–8.0 mm) size classes, proportions of these were between 19–40% (mean 28%) and 8–23% (mean 16%), respectively. Regarding wing morphology, the proportions of threatened species were rather even among the categories. In this respect, wing morphology was not clearly related to species being threatened, except for the slight tendency of wing-polymorphic species being proportionally more frequently threatened in Denmark and brachypterous species in Norway. Regarding shadiness associations, open-area species included proportionally slightly more threatened species than did species of very shady habitats or shadiness generalists. Regarding moisture associations, species associated with very dry habitats included proportionally more threatened species (17–43%) than wet-habitat species (14–29%) or moist/dryish-habitat species (5–26%).

To the extent one can generalise from these figures, in northern and western Europe (see also Casale and Busato 2008 for southern Europe) particular attention should be paid to large carabids, species associated with very dry, open habitats (e.g. sand dunes, heathlands and calcareous meadows; see national Red Lists) and water-associated species (e.g. freshwater stream specialists and salt-marsh species; see national Red Lists). However, as is evident from the variation in percentages presented in Table 3, particular targets of conservation and management (habitat types and species) should vary from one area to another. Below, research-based evidence on how to protect these carabids by the application of conservation management is reviewed.

**ii. Habitat selection for conservation efforts.** Undisturbed mature ecosystems, particularly nature reserves, are vital for the conservation of many carabid species (e.g. Desender 2005; Skłodowski 2006). Also edge habitats and habitat mosaics may be important for carabid conservation (e.g. Kotze 2000; Falke et al. 2000; Hatteland et al. 2005; Andorkó and Kádár 2006). The scarcity of certain habitat types has increased the need for active maintenance of remaining patches. For example, Bérces et al. (2008) used field and museum data, original research and communication among entomologists, and showed that *Carabus hungaricus* can best be protected by active management of open meadows. Due to the on-going loss of natural and semi-natural areas and the intensification of agricultural practices in many countries, also particular anthropogenic habitats have become important for carabid conservation. Examples include roadside verges, former agricultural fields, urban waste-grounds, and sand and gravel pits (Plachter 1986; Eversham et al. 1996; Telfer and Eversham 1996; Schwerk 2000; Versteirt et al. 2002; Koivula and Kotze 2005).

**iii. Habitat connectivity.** Habitat-patch isolation and fragmentation may be of major concern for carabids (De Vries 1994; Kinnunen et al. 1996; Noordijk et al. 2006; Hendrickx et al. 2009). A number of means of reducing the impact of fragmentation have been suggested (Vermeulen et al. 2002). For forested environments, Terlutter (1990) discovered for *Carabus auronitens* two different gene flows from two old forest remnants into a recent, regenerated forest-field mosaic. Later on, Petit (1994) underlined the importance of hedgerow networks for forest carabid assemblages. However, more recent hedges may be sub-optimal for this purpose (Thiele 1971; Gruttke 1994). For open areas, on the other hand, Vermeulen and Opsteeg (1994) showed that roadside verges might be used either as habitat or as movement corridors connecting heathland patches. Both purposes may be served by roadsides, as shown by (Koivula (2002a, 2005) for Finnish forest roads and Noordijk (2009) for highway verges in the Netherlands. Moreover, Vermeulen and Spee (2005) stressed the importance of source habitats for nature restoration sites. Hence, the remaining patches of natural habitat, corridors of similar, often man-made environments, and artificially created, larger patches may together form an efficient patch network for carabid conservation.

**iv. Habitat management.** Because many important natural processes (wildfire, flooding, wind, grazing and insect outbreaks) are effectively prevented in many areas, particularly in urban environments, active maintenance is considered necessary to preserve certain vegetation types. Carabids respond varyingly to these efforts (Versteirt et al. 2002; Cuesta et al. 2006; Taboada et al. 2006a). The effects of grassland management were discussed in depth by Rushton et al. (1990) who found that some species avoid intensively managed sites, some are favoured by these, while others showed intermediate or no detectable responses. Similarly, Blake et al. (1996) showed that vegetation management in wildflower meadows resulted in a decrease in large species and an increase in xerophilous species, while species characteristic of areas with ‘natural’ conditions were absent. Like mowing, grazing also profoundly affects carabids: its intensity determines assemblage composition (McFerran et al. 1994). Cole et al. (2006) showed that intensive grazing decreases the abundance of large *Carabus* species more than less intensive grazing. These studies indicate that variation in management leads to variation in carabid beetle assemblages. In riparian environments, Fuellhaas (2000) showed that raising the water-table level increases the number of hygrophilic species. Främbs (1990) studied regenerating peat bogs and found that although carabid diversity increased, the peat-bog specialist *Agonum ericeti* remained absent. Drees et al. (2007) argued that this might be related to habitat quality, in this case the lack of peat-producing vegetation.

**Figure 4.**
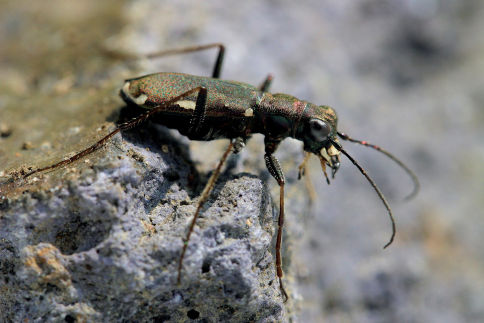
*Cylindera germanica* (Photo by Jinze Noordijk)

To summarise, (i) Carabid conservation should give special attention to very large species, and species associated with both very wet and very dry, exposed conditions, (ii) Old and undisturbed natural areas are important for many specialists, but conservation of pioneer or open-habitat species can be realised in many anthropogenic areas as well (Fig. 4), (iii) Fragmentation potentially isolates local populations, but its effects can be decreased by maintaining large, inter-connected areas, corridor networks, and designing restoration areas near potential source areas, and (iv) Guidelines for active management of carabid habitats are difficult to draft, as some species respond negatively to any disturbance, including conservation management. However, many species urgently need small-scale management that keeps habitats constantly at some preferred successional phase; most of these species are subject to severe stress in modern, fragmented landscapes.

The conservation of carabids and their habitats is far from perfect. This issue is complicated by the fact that, due to these beetles’ mobility, occupation of varyingly sized habitat patches, varying degrees of specialisation, and development through numerous developmental phases, their ecological requirements vary in time and place. As habitat patches of carabid assemblages usually include several vegetation types and/or physical structures, a conservation approach targeted for maintaining only particular vegetation types or high plant diversity may not always be appropriate for the conservation of arthropod assemblages (Panzer and Schwartz 1998; Dennis et al. 2007). Moreover, the high number of carabid species, each with specific demands, makes it difficult to define a single conservation strategy. Protection of whole landscapes with mosaics of distinct habitat types may prove efficient for carabid conservation. Simultaneously, the natural variety of successional stages should be conserved, as particular stages can be crucial for certain species (cf. Niemelä et al. 2007). Moreover, some species – such as *Amara plebeja* (van Huizen 1977) – possibly require more than just one habitat type and/or successional stage to persist in a landscape (‘landscape species’; Szyszko^ 2004^; Szyszko et al. 2011; Axel Schwerk, pers. comm.). Habitat patches within these mosaics should include particular structures, such as micro-relief, patches of bare sand, stony patches, small water bodies, heaps of decaying plant material, and dead wood, features that are often of no special importance for plants and vertebrates and therefore often ignored if conservation management is based on vegetation data alone. Thus, a broad landscape approach, supplemented by these small-scale structures, may produce good results for the conservation of carabid beetles (Kirby 1992; New 1995, 2010; Samways 2005, 2007; Haslett 2007).

## 8 Landscape ecology

How carabid beetles perceive space may influence habitat selection, home ranges, the dispersal of individuals and the dynamics and distributions of populations. Furthermore, the amount, extent and spatial arrangement of suitable habitats within a landscape (i.e. landscape composition and configuration) may affect long-term population persistence. Thus, although the spatial distribution of carabid beetles may be primarily determined by microhabitat conditions and biotic interactions at the local scale, identifying general patterns of carabid responses to landscape features may help us to understand how species, functional groups and assemblages effectively distribute, and to predict how they will cope with current and future land-use and climatic changes. As such, the spatial context related to a species’ distribution patterns is an essential component when studying how global changes affect carabid species conservation.

Over the last 40 years, investigations of ground beetle landscape ecology have demonstrated that landscape features influence not only the spatial distribution of these beetles, but also their population dynamics (Matalin 1997c; Bommarco 1998) and genetic structure (Brouat et al. 2003; Keller et al. 2004; Desender et al. 2005; Sander et al. 2006). From the late 1950s until the mid 1970s, contributions to carabid beetle ecology aimed at characterising the structure and composition of communities occurring in specific types of landscapes, which were, at that stage, considered as homogeneous entities (e.g. forested vs. open landscapes; see Thiele 1977). Research developed in the 1980s and 1990s confirmed the significance of heterogeneity within landscapes and thus addressed the role of singular landscape elements or habitat types for the carabid fauna in a variety of either natural or highly-modified and simplified landscapes. In tests of the application of the theory of island biogeography (MacArthur and Wilson 1967) to carabid communities, it has been shown that local communities are not simply a passive random sample of the regional species pool, but that species are filtered according to the association of their life history traits to habitat quality, configuration and biotic interactions (e.g. Ranta and Ås 1982; Niemelä et al. 1985; De Vries et al. 1996). Studies that have looked for an island effect in carabid assemblages of patches of terrestrial habitats have generally concluded that such patches are not sufficiently isolated to represent islands, due to the strong dispersal capacity of many carabid species (Davies and Margules 1998; Magura et al. 2001; Brose 2003). Studies have also been conducted on carabid assemblages of real islands, and these too have concluded that a simple species-area relationship explains the differences in carabid species richness between islands of different size better than distance from mainland populations (Kotze and Niemelä 2002; Zalewski^ 2004^).

In recent literature, studies on the importance of the landscape context in determining the occurrence of carabid species based on different aspects of landscape composition, configuration, connectivity, history, land-use type and intensity have proliferated (e.g. Purtauf et al. 2004; Bräuniger et al. 2010; Gardiner et al. 2010; Nabe-Nielsen et al. 2010; Woodcock et al. 2010). Many studies analysed the influence of the landscape context on overall carabid beetle activity density and species richness, often finding no statistically significant effect. Mostly, changes in landscape features have been related to shifts in carabid species composition, and variations in the activity density of individual species and ecologically meaningful groups (e.g. Niemelä^ 2001^; Kotze and O’Hara 2003; Niemelä et al. 2007; Niemelä and Kotze 2009).

Agricultural landscapes in particular, driven by daily, seasonal and annual fluctuations, soon became the subject of many carabid beetle surveys, followed by an extensive number of publications to date (e.g. Kinnunen et al. 2001; Holland 2002). In general, the basic composition of the carabid fauna of agricultural mosaic landscapes appears to be surprisingly similar across countries (Luff 2002), dominated by eurytopic species, which are highly tolerant to disturbance. However, the size, amount, isolation and spatial arrangement of agricultural patches, the composition of the arable mosaic, as well as the occurrence of permanent landscape elements (e.g. hedgerows, field margins, natural woodlands and grasslands), affect carabid beetle assemblages (Kinnunen et al. 1996, 2001; Burel et al. 1998; Petit and Usher 1998; Fournier and Loreau 2001; Millán de la Peña et al. 2003; Aviron et al. 2005; Purtauf et al. 2005; Griffiths et al. 2007; Hendrickx et al. 2007; Saska et al. 2007).

In forest ecosystems, natural and anthropogenic disturbances create a dynamic mosaic of successional habitat patches for carabids (e.g. Bouget and Duelli 2004 for windstorm disturbance). Each forest successional stage is characterised by a specific carabid assemblage, in terms of species composition as well as ecological group composition, with the greatest differences between early and advanced stages (e.g. Szyszko 1990; Niemelä et al. 1996; Butterfield 1997; Koivula et al. 2002; Du Bus de Warnaffe and Lebrun 2004; Richard et al. 2004; Magura et al. 2006; Taboada et al. 2008). Changes in population dynamics and morphological traits also take place through succession (e.g. Szysko et al. 1996 for *Pterostichus oblongopunctatus*, Table 4). Changes in the carabid fauna are possibly correlated with the amount of carbon accumulation in the forest system, i.e. in the wood, litter and mineral soil (Szyszko 2010; Szyszko et al. 2011). The increase of carbon in the mineral soil is related to the decomposition of litter by the macrofauna. For pine stands in Poland, Szyszko (1986a) demonstrated that biomass of the macrofauna is correlated with parameters of the carabid fauna, such as species number and Mean Individual Biomass (MIB). MIB increases as succession progresses (Szyszko 1986b; Szyszko et al. 2000; Szyszko^ 2004^), suggesting that this measure functions as a good indicator of the state of succession (see *Bioindicators* above). The rate at which species composition changes during succession and the successional trajectory followed by the carabid assemblages depends on environmental conditions, such as soil properties (Szyszko 1986b, 1990; Schwerk 2008), dominant tree species (Du Bus de Warnaffe and Lebrun 2004), and the type of disturbance that initiated the succession (Du Bus de Warnaffe and Lebrun 2004). Indeed, the larger the newly-created gap is and the fewer trees retained, the more severe the perturbation for carabid assemblages (Koivula 2002b following timber harvest; Bouget 2005 and Skłodowski and Garbalińska 2010 following windthrow gap). As a consequence, the maintenance of a variety of successional phases of the forest cycle results in increased heterogeneity at the landscape level and, therefore greater regional carabid diversity (e.g. Mullen et al. 2008; Taboada et al. 2008). Thus, the effects of forest landscape features on the carabid fauna have also been extensively addressed as regards to landscape heterogeneity, the occurrence, composition and spatial configuration of either natural or human-modified habitats (e.g. proportion of deciduous vs. coniferous forests, age and extent of exotic plantations, forest edge density and permeability), the role of particular landscape elements (e.g. retention tree groups), and the landscape context resulting from historical and/or recent management practices (Koivula et al. 2002; Bouget 2004; Barbaro et al. 2005; Matveinen-Huju et al. 2006; Taboada et al. 2006b; Barbaro et al. 2007; Niemelä et al. 2007; Fuller et al. 2008; Pawson et al. 2008; Barbaro and van Halder 2009).

**Table 4.** Changes in carabid fauna, interaction groups and populations of *Pterostichus oblongopunctatus* with changes in habitat (according to Szyszko et al. 1996, reprinted and modified with permission from Aarhus University Press).

*Comparatively early stage of succession*				*Comparatively late stage of succession*
Carabidae fauna				
low state of development of fauna	→			high state of development of fauna
high number of species	→			low number of species
small individuals	→			large individuals
low mean individual biomass (MIB)	→			high mean individual biomass (MIB)
Interaction group of *Pterostichus oblongopunctatus*				
long period of activity	→			short period of activity
long survival of adults	→			short survival of adults
complicated age structure	→			simple age structure
small individuals (imago)	→			big individuals (imago)
high proportion of males	→			high proportion of females
low number of eggs in ovaries	→			high number of eggs in ovaries
high number of eggs laid?	→			low number of eggs laid?
good food situation for adults?	→			bad food situation for adults?
bad food situation for larvae?	→			good food situation for larvae?
unable to fly?	→			able to fly?
uneconomic life strategy	→			economic life strategy
Populations of *Pterostichus oblongopunctatus*				
asynchronously fluctuating interaction groups	→	synchronously fluctuating interaction groups	→	asynchronously fluctuating interaction groups
low probability of high fluctuations of numbers	→	high probability of high fluctuations of numbers	→	low probability of high fluctuations of numbers
resistant population	→	not very resistant population	→	resistant population

Much effort has been devoted to investigating how carabid beetles are distributed in fragmented landscapes and insular environments (for reviews, see Niemelä^ 2001^ and Kotze 2008, respectively). Carabid responses to fragmentation depend on the geographical context, are species specific and, to a great extent, relate to species’ life history traits and habitat associations (e.g. Koivula and Vermeulen 2005; Gaublomme et al. 2008). In a fragmented landscape context, mobility is crucial for persistence, especially for specialist and scarce species (De Vries 1994; De Vries et al. 1996). In general, good dispersers and abundant species are expected to maintain populations in small and isolated patches through recolonisation of empty patches, whereas poor colonisers and scarce species may not be able to do so (Den Boer 1977; Niemelä^ 2001^). Hostile types of matrix or linear elements in the landscape can act as dispersal barriers for specialist species. For instance, some forest species are reluctant to cross highways (Mader 1984; Koivula and Vermeulen 2005) or open habitats (Plat et al. 1995; Riecken and Raths 1996), while other stenotopic species effectively move along hedgerows (Burel 1989; Plat et al. 1995; Charrier et al. 1997) and roadside verges (Vermeulen 1993, 1994; Vermeulen and Opdam 1995), which function as movement corridors for such species. Dirt roads in forested landscapes may serve as dispersal corridors for open habitat species (Koivula 2002a), and roadsides overgrown with poplars have been suggested to serve as corridors for forest species with low dispersal power (Dymitryszyn et al. 2003). Attempts to improve the connectivity of landscape elements by means of corridors may have contrasting effects on different carabid species according to their habitat requirements and, hence, new approaches regarding this matter are now under evaluation, such as semi-open corridors (Eggers et al. 2010) and innovative, small scale forest harvesting techniques (Koivula et al. 2002, see also *Carabid conservation, protection and habitat management* above).

Further studies have investigated the responses of ground beetles to anthropogenic or human-modified landscapes and urban environments (e.g. Czechowski 1982; Klausnitzer and Richter 1983; Šustek 1987, 1992; Niemelä et al. 2002). These investigations have identified distinct sets of species associated with the urban cores or city centres (but see Niemelä et al. 2002). However, for a considerable number of these species, urban populations may be dependent on recruitment from populations in the urban periphery (Klausnitzer and Richter 1983). The possible effects of urbanisation on carabid population genetics (i.e. genetic diversity and differentiation) remain unclear (Desender et al. 2005). In general, the overall abundance and species richness of carabids decrease with increasing urbanisation (Niemelä and Kotze 2009; but see Magura et al. 2010). Also, large species tend to be relatively scarce in urban habitats, resulting in a decline in average body size in urban areas compared to less disturbed ones, both for forest assemblages (Niemelä et al. 2002; Ishitani et al. 2003; Sadler et al. 2006; Elek and Lövei 2007) and those of open habitats (Czechowski 1982; Šustek 1987; Venn 2007). Flightless species also tend to be relatively scarce in urban assemblages (Venn et al. 2003; Sadler et al. 2006). Other responses detected in carabid assemblages (either in the proportion of species or the number of individuals) to urbanisation include (i) a decrease in species with restricted geographical ranges, along with the enhancement of those distributed over broad ranges; (ii) a decline in oligotopic, stenotopic and specialist species, whilst eurytopic, polytopic and generalist ones increase; (iii) a decrease in forest species and associated increase in open habitat species; (iv) an increase in xerophilic and mesohygrophilous species at the expense of more hygrophilous species; and (v) an increase in omnivorous species and a corresponding decrease in zoophagous species. Whilst stenotopic and specialist species generally decline with increasing urbanisation, some extremely harsh urban habitats accommodate these species, such as populations of *Amara equestris* in central reservations of a busy ring road in Helsinki, Finland (Koivula et al. 2005). In fact, Eversham et al. (1996) reported that more than 35% of Britain’s rare and scarce carabids are to be found from manmade sites, *Omophron limbatum* and *Dyschirius obscurus* exclusively so. Subsequent to numerous urbanisation studies from single cities, the GLOBENET project (Niemelä et al. 2000; http://www.helsinki.fi/science/globenet) was established to apply a standard urbanisation gradient approach in cities across the globe (nine cities located in Europe, Japan and Canada). The main findings indicated that the carabid fauna of urban forested habitats display uniform patterns of response to the degree of urbanisation of the ‘concrete’ matrix (Niemelä and Kotze 2009; Magura et al. 2010).

In the BIOASSESS project (http://www.nbu.ac.uk/bioassess), global patterns in carabid responses to a land-use intensity gradient from old-growth or unmanaged forests to arable crop-dominated landscape across ten countries, have so far reported effects on overall species richness, number of individuals, ecological groups and species composition (Grandchamp et al. 2005; Schweiger et al. 2005; Vanbergen et al. 2005, 2010; Hendrickx et al. 2007; Martins da Silva et al. 2008). Similarly, changes in landscape structure over time (i.e. landscape history) have been addressed when investigating carabid population declines or range-size modifications in human-altered landscapes (Turin and Den Boer 1988; Desender et al. 1994b; Petit and Burel 1998; Kotze and O’Hara 2003). Additionally, these investigations have related contemporary distribution patterns of carabid endemism, rarity and habitat specialisation across landscapes to landscape history.

Even now, the spatial scale at which carabid beetles relate to resources across landscapes is not completely understood. Future studies should accomplish multiscale approaches that consider a wide range of fine and coarse grains at which each carabid species may perceive the landscape, depending on its mobility and body size (e.g. Burel et al. 2004; Aviron et al. 2005; Janssen et al. 2009). The spatial distribution of carabid species in a given landscape is nearly always aggregated at some scale (see e.g. Niemelä et al. 1996; Thomas et al. 2002), which suggests that spatial autocorrelation should always be taken into account (Barton et al. 2009). Additionally, the use of multiple habitats by carabid species in mosaic heterogeneous landscapes (e.g. for feeding, reproducing and overwintering; van Huizen 1977), and the importance of particular habitat combinations for a species’ survival at the landscape level remain unclear (Barbaro et al. 2007). Moreover, since many of the reported carabid responses to landscape features are species specific, more attention should be devoted to the individual species level, and not only for species of present conservation concern but also for common and widely distributed species, as well as to the ecological group level. Nonetheless, sampling strategies that avoid confounding effects are needed to clearly assess the respective weights of local and landscape factors and, at the landscape scale, the respective importance of composition and configuration on a species’ survival. Indeed, experimental landscapes (Davies and Margules 1998) or mensurative experiments (Hurlbert 1984) would be useful to disentangle these gradients that tend to be naturally correlated (Niemelä^ 2001^; Fahrig 2003). Attention should also be paid to discrepancies between carabid species’ responses to landscape features across countries, possibly denoting that the impact of landscape structure on a particular species is likely to differ over its distribution range. Finally, in the current context of continuous landscape transformation, more emphasis should be given to the role of newly created habitats and abandoned areas across countries regarding carabid distribution, as well as to singular expanding elements and surrogate habitats, such as golf courses or private gardens in urban environments (Tanner and Gange 2005; Saarikivi et al. 2010), roads (Koivula and Vermeulen 2005; Melis et al. 2010; Yamada et al. 2010), power lines (Hollmen et al. 2008) and biomass crops (coppice with short and very-short rotation) in either open or forested landscapes. Eventually, potential mechanisms could be investigated by confronting the empirical data with models of dispersal and survival in heterogeneous landscapes (see e.g. Vermeulen and Opsteeg 1994; Pichancourt et al. 2006).

## 9 Concluding remarks

Carabids are among the most species-rich families of beetles, which has made them a natural focus of entomological research. Carabidologists are busy studying this evolutionarily successful group at several levels, from sub-cellular to supra-individual. Indeed, from the discovery of a pH receptor on the antennae of carabid beetles (Merivee et al. 2005; Milius et al. 2006) to cross-continental, landscape related research (Niemelä and Kotze 2009; Magura et al. 2010; Vanbergen et al. 2010) “carabidologists do it all”. They are helped by a reasonably solid taxonomy, even if evolutionary relationships are still undetermined.

Carabidology has contributed to several prominent ecological theories, including metapopulation theory (pioneering work by Piet den Boer and colleagues), and provides one of the best examples of a consistent, systematic study of the effects of urbanisation on biodiversity (Niemelä et al. 2002, and subsequent studies). These somewhat ad hoc examples are still powerful in the argumentation to encourage the use of carabids in ecological, evolutionary and behavioural studies.

Even from a subjective summary as this article admittedly is, it is obvious that carabids have contributed in a major way to our understanding of invertebrate adaptations, phylogeny and ecology. Accepting Hutchinson’s analogy that on the world stage an ecological play is being played out in the evolutionary theatre (Hutchinson 1965), watching and describing the peculiarities of one of the star players, ground beetles, will certainly advance our understanding of nature. In an age in which the earth is dominated by humans, this will provide important knowledge on how to maintain the richness of life on Earth, and with it, extend the lifespan of our own species.
